# Dissection of Symbiosis and Organ Development by Integrated Transcriptome Analysis of *Lotus japonicus* Mutant and Wild-Type Plants

**DOI:** 10.1371/journal.pone.0006556

**Published:** 2009-08-07

**Authors:** Niels Høgslund, Simona Radutoiu, Lene Krusell, Vera Voroshilova, Matthew A. Hannah, Nicolas Goffard, Diego H. Sanchez, Felix Lippold, Thomas Ott, Shusei Sato, Satoshi Tabata, Poul Liboriussen, Gitte V. Lohmann, Leif Schauser, Georg F. Weiller, Michael K. Udvardi, Jens Stougaard

**Affiliations:** 1 Centre for Carbohydrate Recognition and Signalling, MBI, Aarhus University, Aarhus C, Denmark; 2 Bioinformatics Research Center (BiRC), Aarhus University, Aarhus C, Denmark; 3 Max-Planck-Institute for Molecular Plant Physiology, Potsdam, Germany; 4 ARC Centre of Excellence for Integrative Legume Research, Genomic Interactions Group, Research School of Biological Sciences, Australian National University, Canberra, Australian Capital Territory, Australia; 5 Kazusa DNA Research Institute, Kisarazu, Chiba, Japan; 6 Plant Biology Division, The Samuel Roberts Noble Foundation, Ardmore, Oklahoma, United States of America; University of Toronto, Canada

## Abstract

Genetic analyses of plant symbiotic mutants has led to the identification of key genes involved in *Rhizobium*-legume communication as well as in development and function of nitrogen fixing root nodules. However, the impact of these genes in coordinating the transcriptional programs of nodule development has only been studied in limited and isolated studies. Here, we present an integrated genome-wide analysis of transcriptome landscapes in *Lotus japonicus* wild-type and symbiotic mutant plants. Encompassing five different organs, five stages of the sequentially developed determinate *Lotus* root nodules, and eight mutants impaired at different stages of the symbiotic interaction, our data set integrates an unprecedented combination of organ- or tissue-specific profiles with mutant transcript profiles. In total, 38 different conditions sampled under the same well-defined growth regimes were included. This comprehensive analysis unravelled new and unexpected patterns of transcriptional regulation during symbiosis and organ development. Contrary to expectations, none of the previously characterized nodulins were among the 37 genes specifically expressed in nodules. Another surprise was the extensive transcriptional response in whole root compared to the susceptible root zone where the cellular response is most pronounced. A large number of transcripts predicted to encode transcriptional regulators, receptors and proteins involved in signal transduction, as well as many genes with unknown function, were found to be regulated during nodule organogenesis and rhizobial infection. Combining wild type and mutant profiles of these transcripts demonstrates the activation of a complex genetic program that delineates symbiotic nitrogen fixation. The complete data set was organized into an indexed expression directory that is accessible from a resource database, and here we present selected examples of biological questions that can be addressed with this comprehensive and powerful gene expression data set.

## Introduction

Legumes constitute the third largest family (Fabaceae) of flowering plants and they are second only to grasses in their economic and nutritional importance. Several legumes, including soybean, common bean and alfalfa, are major crops producing protein and oil for food and feed. A key trait of legumes is the competence for symbiotic nitrogen fixation, which is the result of an intimate relationship with a group of soil living bacteria collectively called rhizobia. Initial signal exchange between the symbiotic partners triggers a plant morphogenetic program leading to the formation of root nodules, inside which bacteria is hosted and which reduce gaseous nitrogen into ammonium. This eliminates the need for nitrogen fertilizer in crop legumes. Not only does the understanding of this mutualistic association hold the key to a better exploitation of a trait important in agriculture, it also provides insights into molecular processes controlling microbe recognition, pathogen defense and plant organogenesis. Providing impetus to legume research, *Lotus japonicus* and *Medicago truncatula* have been adopted as the principal model legumes. Their diploid genome, short life cycle, susceptibility to *Agrobacterium* transformation, and other favourable biological features distinguish *Lotus* and *Medicago* from the crop legumes, and these features are the foundations for implementation of the current genetics and genomics approaches.

One of the goals of research on symbiotic nitrogen fixation is to identify and assign a function to all genes acting in the pathways leading from bacterial recognition to development of a new plant organ, the nodule, and to determine how they interact. Legumes encode all functions necessary for nodule development, as demonstrated by the spontaneous development of nodules in certain legume mutants grown axenically [1], [2]. Thus, by studying plant genes alone, the genetic disposition for root nodule development can be elucidated. In recent years, several symbiotic mutants impaired at different stages of nodulation and mycorrhization have been characterized, and key genes have been identified using genetic approaches (reviewed in [3]). Bacterial signals, called Nod-factors are perceived in *Lotus* by the NFR1 and NFR5 receptor kinases, and both receptors are required for the host plant to initiate infection and nodule organogenesis [4]–[6]. Further downstream, comparable gene products from *Lotus* and *Medicago* contribute to the signal transduction pathway shared with mycorrhizal fungi [7]. SYMRK/NORK/DMI1, a leucine rich repeat receptor kinase [8], [9], CASTOR and POLLUX/DMI2, a putative cation channel(s) [10], [11], as well as the nucleoporins NUP133 and NUP85 [12], [13] are all required for induction of calcium spiking, a rapid physiological response in root hairs detected after Nod-factor application [14]. Calcium spiking is believed to be interpreted by a calcium calmodulin dependent kinase, CCAMK, which acts together with the CYCLOPS protein to mediate downstream responses [1], [15]–[18]. Putative transcriptional regulators NIN, NSP1, NSP2 and members of the ERF transcription factor (TF) family mediate bacterial infection at the root epidermis and nodule organogenesis in the root cortex [19]–[23].

As a result of large-scale genome sequencing efforts, lists of genes with unknown function are expanding rapidly, and, consequently, there is a need to apply high-throughput approaches for rapid characterization of genes. Plant genomics tools have matured in the non-legume *Arabidopsis*, and array-based transcript profiling has become efficient and widely used. One major outcome is the expansion of data resources, like the AtGenExpress project, that hold gene expression information for almost all *Arabidopsis* genes.

More recently, large scale sequencing in legumes provided the information for array design and production, and several studies of rhizobial associations have been conducted using EST-based macro- and micro-arrays in both *Medicago* [24]–[27] and *Lotus* [28]–[31]. These studies have identified legume-specific genes, rhizobia-inoculation-responsive genes (early nodulins) and genes functioning in mature nodules (late nodulins).

The *Lotus japonicus* genome sequencing project [32] allowed the design of an Affymetrix GeneChip® containing more than 52,000 *Lotus* probe sets (collection of probes on the GeneChip® designed to represent a transcript), representing all known and predicted open reading frames (ORFs) in the available 315 Mb gene space. By coupling the power of nearly full genome coverage with organ-specific sampling we were able to unravel the transcriptional signatures caused by genetically arresting nodule development at different stages. We present these data as a web-accessible resource containing gene expression data covering several aspects of legume development and symbiotic biology.

## Results and Discussion

### The *Lotus* Transcript Profiling Resource

The *Lotus japonicus* GeneChip® contains 52,749 *Lotus* and 8,710 *Mesorhizobium loti* (*M. loti*) derived probe sets, each representing a known or predicted open reading frame (ORF) or miRNA. We used this platform to profile the transcriptome of roots, root nodules, stems, leaves and flowers and to identify *Lotus* genes defining the identity of the main legume organs. A special effort was devoted towards profiling the symbiotic interaction with the microsymbiont, *M. loti*, and development of root nodules. The determinate nature of root nodule development in *Lotus* lends itself to this approach since the developmental stages occur sequentially. Eight *Lotus* mutants arrested at different stages in the symbiotic process and a root nodule developmental time-series was profiled in order to define important regulatory checkpoints. To complement this approach we included a selection of tissues from shoot and root in order to evaluate *Rhizobium*-legume interactions and shoot-root communication, which is known to be essential for controlling nodule number [33]. Finally, we measured the effect of four different treatments, nitrate, Nod-factor, inoculation with an *M. loti nodC* mutant, and inoculation with the *M. loti* wild-type on the transcriptome. To minimize biological variation, plants were grown under the same conditions and all samples from a particular condition/treatment were harvested at the same time of the day. To strengthen the universal validity of the results, biological replicates were obtained by growing all plants in three spatially and temporally separated batches, with all conditions represented in each batch. By setting up three experiments and three harvests, we ensured that all observed differences are reproducible between experiments, and not just between samples harvested in a single experiment, which is a commonly used strategy. Altogether, 38 sets of transcriptional profiles were obtained from roots, nodules, shoots, leaves, stems and flowers exposed to a variety of treatments (Table 1, Figure S1).

**Table 1:** All conditions profiled.

Condition	Samples	Genotype	Treatment	Days after inoculation	Tissue
T1	3	*nfr1*	R7A	1d	SZ
T2	2	*nfr5*	R7A	1d	SZ
T3	3	*nin*	R7A	1d	SZ
T4	3	*nup133*	R7A	1d	SZ
T5	3	*WT*	R7A	1d	SZ
T6	2	*WT*	purified Nod Factor	1d	SZ
T7	3	*nfr1*	–	–	SZ
T8	3	*nfr5*	–	–	SZ
T9	3	*nin*	–	–	SZ
T10	3	*nup133*	–	–	SZ
T11	3	WT	–	–	SZ
T12	3	WT	–	–	Root Tip
T13	3	WT	R7A	1d	Whole Root
T14	3	WT	R7A nodC	1d	Whole Root
T15	2	WT	R7A	3d	Whole Root
T16	3	WT	R7A	3d	Shoot
T17	2	*har1*	R7A	3d	Whole Root
T18	3	*har1*	R7A	3d	Shoot
T19	2	*har1*	–	–	Whole Root
T20	2	*har1*	–	–	Shoot
T21	3	WT	–	–	Whole Root
T22	3	WT	–	–	Shoot
T23	3	WT	5 mM Nitrate	–	Whole Root
T24	3	WT	5 mM Nitrate	–	Shoot
T25	2	*cyclops*	–	–	Whole Root
T26	3	*sen1*	–	–	Whole Root
T27	2	*sst1*	–	–	Whole Root
T28	1	*cyclops*	R7A	21d	Root+Nodule
T29	3	*sen1*	R7A	21d	Nodule
T30	3	*sst1*	R7A	21d	Nodule
T31	3	WT	R7A	21d	Nodule
T32	3	WT	R7A	21d	Root+Nodule
T33	3	WT	R7A	14d	Nodule
T34	3	WT	R7A	7d	Root+Nodule
T35	3	WT	5 mM Nitrate for 6 weeks	–	Whole Root
T36	3	WT	5 mM Nitrate for 6 weeks	–	Leaf
T37	3	WT	5 mM Nitrate for 6 weeks	–	Stem
T38	3	WT	5 mM Nitrate for 13 weeks	–	Flower

R7A: *Mesorhizobium loti* strain R7A, SZ- susceptible zone.

Annotation of the *Lotus* genome and thus the assignment of gene identification indices is not yet fully completed. When describing expression patterns, a gene or an “ID” (shorthand for “identifier”) therefore refers to a predicted transcript whose sequence guided the design of a Probe Set on the *Lotus* Affymetrix array. Unless otherwise stated, a False Discovery Rate (FDR) corrected p-value≤0.05 was used as the criterion for significance in all statistical analyses, in combination with an |M|≥1 filter, where M is the log2 ratio of average expression values from any two conditions.

A database holding raw and normalized expression data, together with information about sample generation and characteristics was created and is publicly available (http://www.brics.dk/cgi-compbio/Niels/index.cgi). This website also features a comprehensive set of tools for mining and presenting the data, such as a tool to visualize the expression profile of one or more genes across conditions, and a pattern matching tool that can be used to identify genes having similar expression profiles. The database is an open and flexible source for extracting and comparing transcript profiles (expression pattern of a transcript across conditions) according to individual research objectives. Furthermore, all gene expression data have been deposited in the ArrayExpress database (http://www.ebi.ac.uk/microarray-as/ae/).

Here we illustrate how this data set can be mined to extract biologically meaningful information using only a fraction of this very large data set.

### *Lotus* transcriptional dynamics during nodule development and onset of symbiotic nitrogen fixation

To thoroughly uncover the root- and nodule- transcriptome regulation during the establishment of symbiotic nitrogen fixation, we used the *Lotus* whole genome array to identify the changes induced at the mRNA level by the microsymbiont *M. loti*. Four distinct developmental stages were chosen. The first stage was one day post inoculation (1 dpi), when early signalling events have been initiated; root hairs curl and entrap bacteria. The next stage was 3 dpi, when the infection process has been initiated, and the first infection threads are visible. At 7 dpi, nodule primordia are formed, and organogenesis is progressing, and at 21 dpi when most of the nodules are mature, symbiosome differentiation is complete and symbiotic nitrogen fixation established. In an attempt to capture the regulation processes in both nodules and the supporting roots we chose to profile transcripts of whole root systems carrying developing or fully developed nodules. First, the transcript profile of each developmental stage was compared to uninoculated roots, and only genes satisfying the previously mentioned criteria (M, FDR) in these contrasts were used in the subsequent comparisons presented here. In the time-course experiment a particular gene was considered stage(s)-specific if the above criteria were met only at that particular developmental stage(s).

The general picture that emerged from comparing uninoculated to inoculated/nodulated roots at the selected time-points was that positive and negative transcriptional regulation primarily occurred at 1 dpi (1015 IDs) and 21 dpi (2930 IDs). A smaller number of genes were stage-specifically regulated at 3 and 7 dpi, corresponding to 283 and 633 IDs, respectively. At 3 and 7 dpi, the majority of stage-specific genes were up-regulated (67% at 3 and 69% at 7 dpi), in contrast to 1 and 21 dpi where a more equal distribution of up-/down-regulated specific genes (44/56% at 1 dpi and 48/52% at 21 dpi) was observed (Figure S2). Surprisingly, only a small number of genes (48 IDs) were regulated throughout the entire developmental time-course. This group includes several nodulins, serpins, expansin, pectinestarases, endo-1,4-beta-D-glucanases and pectate lyase. Up-regulation of the latter enzymes involved in cell wall extension and loosening, is in good agreement with the general belief that modification of cell walls is important for nodule growth and expansion, for infection thread initiation and elongation towards the primordia, and for release of rhizobia into nodule cells. Several of these cell wall modifying genes were also found highly induced during the development of nematode syncytia [34]–[36].

Several genes encoding putative transcriptional regulators (TFs), like *Nin*, *Nsp1*, a GRAS-type TF highly similar to *Nsp2* (*Nsp3*), *ARR8* and others (Myb-like, Wuschel-like homeobox, ERF-AP2 and CCAAT) were also found to be regulated during nodule development. *Nin* [20] maintained a high expression level from 1 to 21 dpi, while *Nsp1* [21] had the highest level at 1 dpi (4 fold up-regulated) followed by a slight decline in the level of induction (1.5 fold up-regulated at 21 dpi), which is in agreement with the previously reported expression pattern. In our data set, the *Nsp2* gene was found to be strongly down-regulated (12 fold at 21 dpi), but no induction was observed at 1 or 7 dpi when the whole root was analyzed. However, looking in more detail at the root susceptible zone (SZ) 1 dpi, a slight up-regulation of *Nsp2* was observed. Interestingly, *Nsp3* was identified as significantly down-regulated throughout the time-course analysis indicating multiple functions of several GRAS-type TFs during nodule development and maintenance (Folder S1).

Induction of known nodulins including *N21*, *N16*, *N26*, *N56* and leghemoglobin was confirmed. Several of these were up-regulated at all stages investigated (*N16* and *N26*), whereas *N21* and leghemoglobin were specifically up-regulated later, at 7 and 21 dpi during nodule development. The sulfate transporter gene, *Sst1*, was highly induced at 21 dpi supporting the reported induction between 7 and 14 dpi [37] (Folder S1).

One of the most prominent types of genes, that were down-regulated specifically at 1 dpi in *Lotus*, is encoding apyrase (3 IDs). Our experimental set-up did not cover the 3 and 6 hours after inoculation at which up-regulation of this gene was observed by Cohn et al. [38], but we do observe the down-regulation at 1 dpi previously observed by Navarro-Gochicoa et al. [39]. Interestingly, given recent data on their involvement in growth, development and signalling this suggests a key role for the control of extracellular ATP during the establishment of symbiosis [40], [41].

Suppression of plant defense mechanisms during nodulation was previously reported [24], [25]. Analysis of the defense related genes revealed down-regulation for several of them at 1 dpi indicating that *M. loti* is recognized as a beneficial microbe. This includes esterase/lipases, disease resistance response protein 206, and TMV resistance protein N, previously found to be involved in defense responses activated during pathogen infections [42], [43]. However, some defense related genes were found up-regulated 1 day after inoculation including those encoding PR1 and thaumatin. Up-regulation of genes encoding serpins (cysteine protease inhibitors), which suppress cell-death [44], was observed at all nodule developmental stages. This implies, that during establishment and development of root symbiosis, cell death processes are inactivated, which might be important for accommodation of the bacterial symbiont within the plant cells (Folder S1).

Phytohormones are known to play an important role during root nodule development [45]–[49]. Application of exogenous ethylene or stimulation of ethylene biosynthesis suppresses nodulation, whereas application of ethylene biosynthesis inhibitors increases nodule number [45]. Antagonism between Nod-factor and ethylene perception has also been shown [46]. One day after application of rhizobia, a significant reduction of ethylene biosynthesis gene-transcripts was detected while transcript levels of the ethylene receptors, which negatively regulates ethylene mediated signalling pathways were found to increase. This effect was reversed at 7 dpi when an ethylene biosynthetic gene was strongly up-regulated along with TFs previously identified to be involved in the ethylene signalling pathway. These observations indicate that a tight regulation of ethylene biosynthesis and its signalling pathway is necessary for proper root nodule development and rhizobial infection.

Initiation of root nodule development involves de-differentiation of cortical cells resulting in cell proliferation and initiation of a new meristem, the root nodule primordium. The plant phytohormone cytokinin has been shown to play an important role in this process [47]–[49]. We found that several histidine kinase encoding genes (putative cytokinin receptors) were up-regulated at 3 and 7 dpi, when nodule primordia develop, and then down-regulated at 21 dpi when mature nodules developed. The earliest cytokinin-related response observed at 1 dpi was the induction of a cytokinin oxidase gene and a strong up-regulation of *ARR8*, which remained at high levels throughout the entire time-course. In *Arabidopsis*, it is hypothesized that ARR8 and ARR9 are key elements in the cytokinin regulation of lateral root development [50]. We found homologs of *ARR9* to be up-regulated at 21 dpi together with several other *ARR*s (*ARR3* and *17*, Folder S1). Their involvement in establishment of nitrogen-fixing symbiosis is an example of general plant developmental genes employed for root nodule development. Components of the brassinosteroid, abscisic acid, gibberellin, auxin and jasmonate signalling pathways were also identified in our time course data as being tightly regulated through-out nodule development, indicating a strong phytohormonal control of the process (Folder S1).

Previous transcriptomic studies performed on *Lotus* and other legume species showed regulation of metabolic pathways during symbiotic nitrogen fixation. Here, we found down-regulation of flavonoid biosynthesis genes, up-regulation of genes for carbohydrate metabolism and a strong up-regulation of genes for amino acid metabolism in mature nodules. Amino acid and ammonium exchange between the plant host and bacteria is important for symbiotic nitrogen fixation, and analyses of our data showed regulation of genes encoding proteins involved in glutamate and asparagine biosynthesis in 21 dpi nodules. Glutamine hydrolase involved in glutamine conversion to glutamate was highly up-regulated together with asparagine synthase and asparatate aminotransferase genes. A strong up-regulation of several peptide transporter genes was also detected, probably reflecting the importance of amino acid transport during nodule development and maintenance of symbiotic nitrogen fixation.

### Root sectors undergo specific transcriptional reprogramming after *M. loti* inoculation

Legume roots are able to recognize rhizobia as symbionts and previous microscopic, and genetic studies performed on different legume species showed that the root sector containing elongating root hairs is the most responsive to the presence of Nod factor producing symbiotic bacteria (reviewed in [51]). We have used the term “susceptible zone (SZ)” for this root segment. In order to have a comprehensive view of *Lotus* whole root reactions to symbiotic bacteria, we analyzed the responses 24 hours after application of *M. loti* wild-type and *M. loti nodC* (mutant unable to produce Nod-factors). Furthermore, we have analyzed the transcriptional changes in the SZ upon application of purified Nod factors and Nod factor-producing bacteria. The transcript profile of the treated whole roots and SZs were compared to the corresponding untreated root sectors. Only genes, satisfying the significance criteria (M, FDR) in these contrasts were used for subsequent analysis. A particular gene was considered root sector and/or treatment-specific if the above criteria were met only at the specified condition(s).

**Figure 1:**
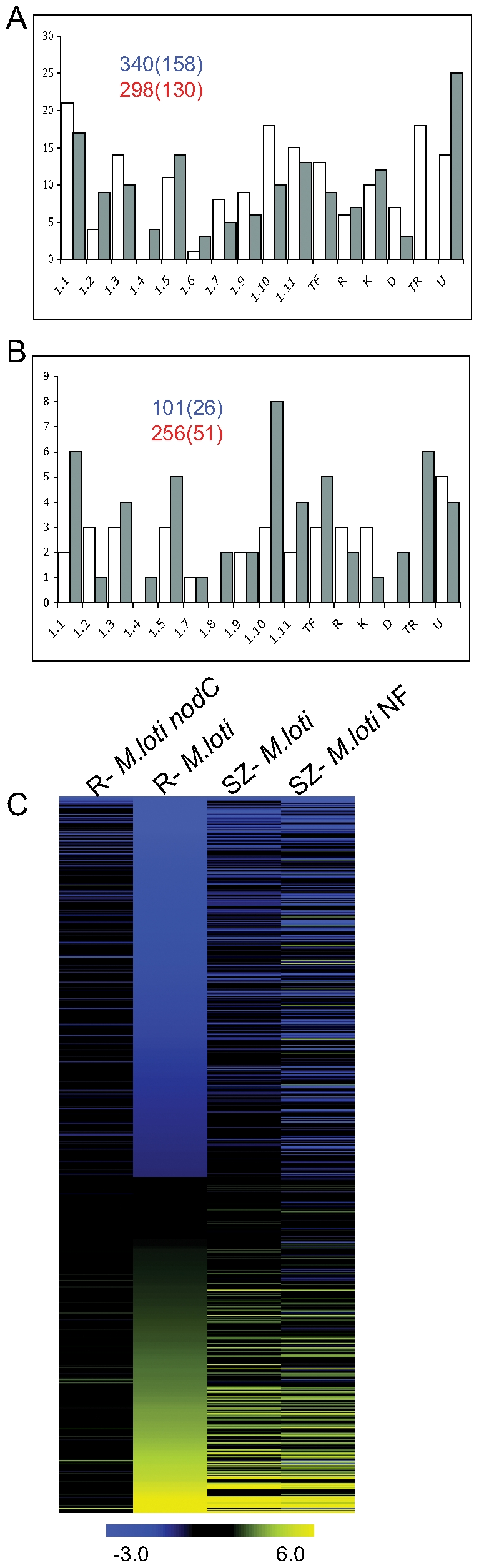
*Lotus* root responses to *M. loti* inoculation. Number of specific IDs down-regulated (white) and up-regulated (grey) at the whole root level (A) and in the SZ (B) 24 hours after *M. loti* inoculation. IDs are organized based on their KEGG annotation (metabolism of: 1.1-carbohydrate, 1.2- energy, 1.3- lipid, 1.4- nucleotide, 1.5- amino acid, 1.6- other amino acids, 1.7- glycan biosynthesis and metabolism, 1.9- cofactors and vitamins, 1.10- biosynthesis of secondary metabolites, and 1.11- xenobiotics biodegradation and metabolism) and their putative annotation (TF-transcription factor, R-receptor, K-kinase, D-defense, TR-transporter, U-unknown). Graph insets show the total number of probe sets down-regulated (blue) and up-regulated (red) in the analyzed tissues with the specific ones in brackets. C) Pattern of the IDs regulated by *M. loti* at the whole root level (FDR≤0.05 and |M|≥1) in the other analyzed tissues and treatment (R-whole root, SZ-susceptible zone, NF-Nod factor)

An interesting feature was observed when the whole root gene expression was compared to the SZ in response to *M. loti* treatment. Although the SZ has the most prominent morphological reaction towards rhizobia, we found that the whole root was more responsive to inoculation than the SZ (638 IDs regulated in the whole root compared 357 in the SZ). Around 50% of *M. loti* regulated genes in the whole root were specific (288 IDs out of 638) (Folder S2). Their tendency was down-regulation, except for those predicted to be involved in energy, nucleotide and amino acids metabolic pathways which were generally found to be up-regulated (Figure 1A). To estimate the extend of dilution effects in this comparison we looked at transcript levels for the *Nin* and *Enod40* genes that were previously shown to be specifically transcribed in the SZ. Both genes showed similar levels of transcriptional activity in the whole root and the SZ indicating a minimal impact from dilution.

We found in the SZ less than 25% of the *M. loti* regulated genes to be specific (77 out of 357 IDs) (Folder S2), and most of them were up-regulated. This trend was clear for genes involved in regulation of metabolic pathways, while those implicated in signal transduction (TFs, receptors and kinases) were regulated in both directions (Figure 1B). This shows that symbiotic bacteria trigger distinct processes in *Lotus* root sectors differing in their developmental status or susceptibility.

Transcriptome analysis of *Lotus* roots exposed to *M. loti nodC* mutant revealed a very limited response. Genes corresponding to only 10 IDs were found significantly regulated, indicating that Nod-factor perception is a prerequisite for most of the subsequent transcriptional responses of the legume root (Figure 1C). On the other hand, Nod-factor treatment led to widespread changes in the *Lotus* transcriptome. Approximately 10% of all genes were regulated (5014 IDs, of which 4551 were specific) (Folder S2). Many of the genes, which were found affected by Nod-factor producing bacteria, were also regulated by the Nod-factor treatment (120 IDs for the SZ and 166 IDs for the whole root) (Folder S2).

Although limited morphological responses are observed on legume roots 24 hours post-inoculation, major changes occur at molecular and cellular levels [51]. In order to understand how different gene classes previously implicated in the early symbiotic events participate in rhizobial induced signal transduction cascades, we identified TFs, receptors and kinases specifically regulated in response to *M. loti* in wild-type *Lotus* roots (Figure S3A to F). In the SZ, a specific set of TFs (8 IDs), receptors and kinases (8 IDs) were responding to rhizobia (Figure S3A and D). Besides these TFs regulated specifically by *M. loti*, an additional number (7 IDs) were found regulated in the SZ by Nod-factor application (Figure S3C). The identified TFs belong to different classes, and included *Nsp2*, which is part of the GRAS family. *Lotus* homologs of *Arabidopsis* TFs required for specification of meristem identity in the aerial parts (MYB17-chr5.CM0148.21 and AGL62- chr5.TM1466.3.1) were regulated in the SZ upon rhizobial inoculation. In addition, a homolog of ANR1 (chr2.CM0177.35.1), a nitrogen-dependent regulator of *Arabidopsis* lateral root development [52], was down-regulated in both rhizobial and Nod-factor treated roots.

Coordination of phytohormone signalling in particular legume root cell layers is required for nodule primordium initiation and nodule number regulation [45], [48]. Our detailed transcriptome analysis of *Lotus* roots points towards a complex hormonal regulation, and genes predicted to encode proteins involved in ethylene, auxin, cytokinin, brassinosteroid, jasmonic acid, and abscisic acid signalling pathways, were regulated by *M. loti* or *M. loti* Nod-factor in the first 24 hours (Folder S2). Both upstream and downstream signalling components of cytokinin pathway were regulated showing that hormonal control feedback loops are set in place (Figure S3G and H). Differences in expression pattern of specific signalling components were found between the whole root and the SZ in the presence of. *M. loti*. The cytokinin receptor *Lhk1*, the downstream response regulator *ARR3* and a cytokinin oxidase gene were up-regulated preferentially in the SZ, while the *ARR8* was up-regulated in the whole root (Figure S3H). Different cyclin-dependent kinase genes were identified to be specifically up-regulated in the two samples; a type D5 in the SZ, and a type D3 in the whole root (Folder S2). The latter type of kinase was previously shown to be important for determining cell number in developing lateral organs [53]. Taken together, these data indicate that mechanisms controlling nodule numbers (cortical cell division foci) may be established within 24 hours after inoculation.

### Symbiotic mutants assist dissecting the sequence of *M. loti* induced transcriptome changes in *Lotus* roots

In order to understand the genetic regulation of gene expression during early symbiosis we profiled the SZ of four symbiotic *Lotus* mutants altered in the initial signalling process induced by *M. loti*: *nfr1*, *nfr5*, *nup133* and *nin*. All four mutants display a non-nodulating, non-infected phenotype, however they differ in their degree of cellular, morphological and physiological responses. *nin* mutant plants, which are mutated in a putative TF, respond with calcium spiking and substantial root hair deformation [20]. *nup133* mutants are affected in one of the nucleopore components, lack calcium spiking, and display limited root hair deformations upon inoculation [12]. *nfr1* and *nfr5* mutant plants, impaired in LysM receptor kinases, are insensitive to rhizobia or Nod-factor application [4], [5]. In our analysis, the transcript profile of mutant and wild-type SZ at 1 dpi was compared to the corresponding uninoculated SZ and only genes that satisfied the criteria (M and FDR) in these contrasts were subsequently used for comparisons between different genotypes. A particular gene was considered genotype(s)-specific if the criteria were met only by the specified genotype(s).

**Figure 2:**
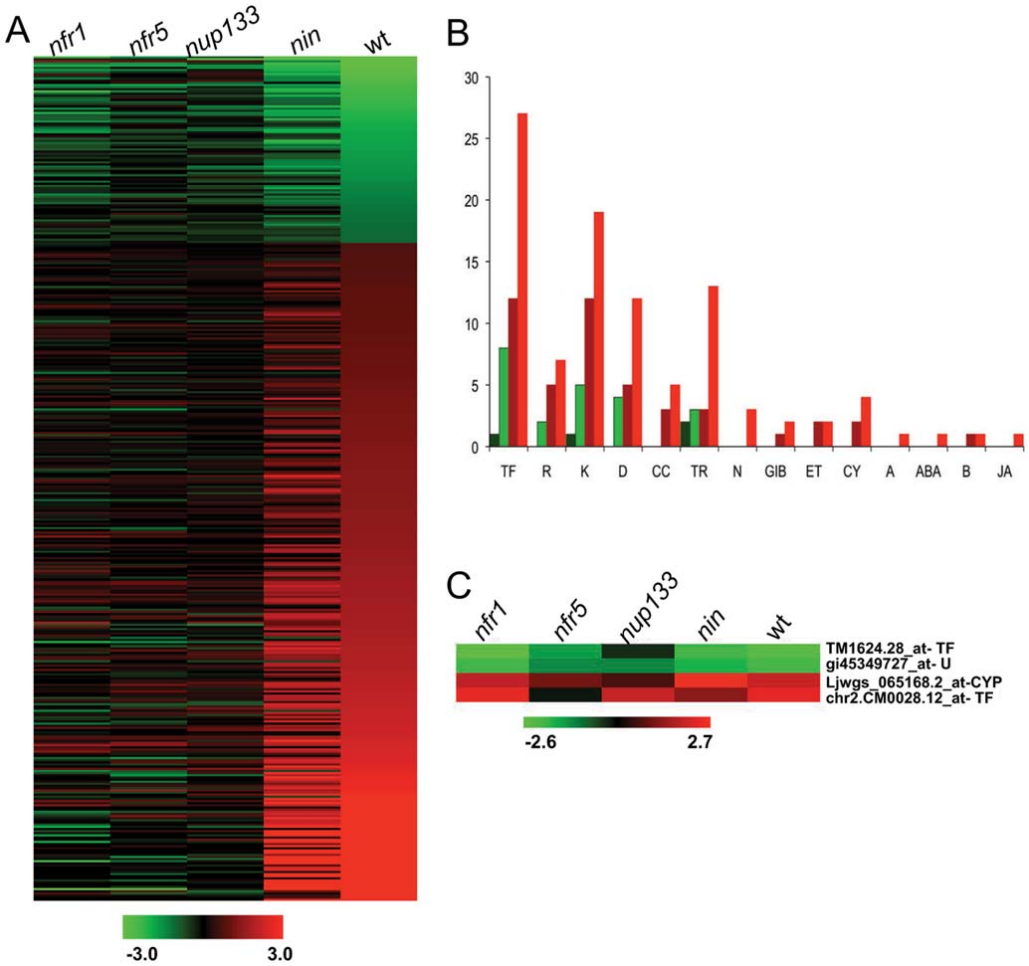
Transcriptome responses induced by *M. loti* in wild-type and mutant *Lotus* plants. A) Pattern of the IDs significantly regulated by *M.loti* in the SZ of wild–type (wt) *L. japonicus*, in the *nfr1*, *nfr5*, *nup133* and *nin* mutants. B) Number of IDs found to be down-regulated in *nin* (dark green) and wt (light green) and up-regulated in *nin* (dark red) and in wt (light red) upon *M. loti* inoculation sorted according to their annotation. C) Pattern of the IDs significantly regulated by *M. loti* in the SZ of wild-type, *nfr1* and *nin* (only Ljwgs_065168.2_at). Their pattern in the *nfr5* and *nup133* mutants is presented along. An FDR corrected p-value≤0.05 and |M|≥1 filter were used as criteria for significance. TF-transcription factor, R-receptor, K-kinase, D-defense, CC-cell division, TR- transporters, N-nodulin, GIB-gibberellin, ET- ethylene, CY- cytokinin, A-auxin, ABA-abscisic acid, B-brassinosteroid, JA-jasmonic acid, U- unknown, CYP- cytochrome P450.

None of the mutants had an *M. loti* induced transcriptome response similar to wild-type plants, showing that the affected genes control key steps in the early signalling pathway (Figure 2A). Nonetheless, of the four mutants, *nin* plants had a transcript profile most similar to wild-type. Around 40% of the genes (159 IDs), which had an altered expression in the wild-type, were regulated in the *nin* mutant (Figure 2A and Folder S3), and many of the signalling processes induced by *M. loti* in the wild-type were also induced in *nin* mutants (Figure 2B and Folder S3). During the first 24 hours of symbiosis, regulation of more than 75% of the genes that were repressed (63 out of 87 IDs), and over 50% of the genes that were induced (160 out of 301 IDs) in the wild-type depended on NIN. This adds to around 100 genes (122 IDs) found to be *nin* mutant specific, summing up to a total of over 300 genes (345 IDs) whose correct regulation depended on NIN (Folder S3). Ethylene and brassinosteroid signalling components were regulated upon inoculation in both *nin* and wild-type while for gibberellic acid and cytokinin signalling, only some of the components were regulated similarly in the two genotypes. None of the genes involved in auxin, abscisic acid or jasmonic acid signalling regulated in wild-type were identified in the *nin* mutant, suggesting that the *Nin* gene acts upstream of these *M. loti* induced hormonal pathways (Figure 2B). Of the TF-encoding genes regulated specifically by *M. loti* or Nod-factor in wild-type SZ (Figure S3A), two were also regulated in *nin*, suggesting a function upstream or independent of NIN. Both are predicted to encode helix-loop-helix TFs with unknown function in *Arabidopsis*. Interestingly, NIN was found not to control its own transcription, and among the two GRAS-type TFs, NSP1 and NSP2, which were previously shown to act upstream of NIN [23], *Nsp2* up-regulation depended on an active *Nin* gene, while *Nsp1* did not (Folder S3).

In the *nfr5* and *nup133* mutants the transcript changes in response to *M. loti* were virtually absent (Figure 2A). A single gene (ubiquitin-conjugating enzyme) was specifically down-regulated in the *nfr5*, and this regulation was also detected in the *nfr1* mutant (see below). In *nup133*, two genes were specifically down-regulated, one encoding a protein with unknown function, the other a Sec5 protein, which in *Arabidopsis* is important for cell morphogenesis [54] (Folder S3). This indicates that root hair morphological changes induced by *M. loti* in *nup133* occur independently of changes in transcript levels or that these were below the detection level at 1 dpi. The occasional nodule formation on the roots of this mutant might be a result of delayed transcriptional changes. These results show that the early changes in transcriptional activity upon *M. loti* inoculation depend on Nod-factor perception mediated by the NFR5 receptor and on molecular transport through nuclear pores (reviewed in [55]) or nucleoporin mediated transcriptional activation [56].

The *nfr1* mutant was more responsive to *M. loti* inoculation compared to *nfr5* and *nup133*. Almost 200 genes (194 IDs) responded to *M. loti* inoculation in *nfr1*. The majority of these genes (184 IDs) were specifically regulated in the *nfr1* mutant and only 4 genes were also regulated in wild-type (Figure 2C and Folder S3).

Overall, analysis of root transcriptional responses to *M. loti* in these five genetic backgrounds revealed that regulation of the vast majority of genes (389 out of 393 IDs), in the wild-type SZ depends on both the *Nfr1* and *Nfr5* genes. Initiation of the signal cascade in response to Nod-factor is therefore most likely mediated by a receptor complex containing both NFR1 and NFR5 or by convergent signalling from two separate NFR1, NFR5 receptor complexes. The remaining four genes regulated in *nfr1* mutants may be under control of NFR5 and indicate independent signalling from this receptor. Identification of many genes, which are specifically regulated in the *nfr1* mutant in response to *M. loti* inoculation, may reflect an independent function that is altered in the absence of NFR1 or alternatively, that NFR1 may control this gene set independently. Altogether, these results are in accordance with the described morphological, physiological and symbiotic phenotypes of the *nfr1*, *nfr5* and *nup133* mutants [4], [5], [12], and our results show that transcriptional changes induced in *L. japonicus* SZ by *M. loti* were dependent on LysM receptor proteins and nucleoporins.

### A cyclopean view of the symbiotic process

**Figure 3:**
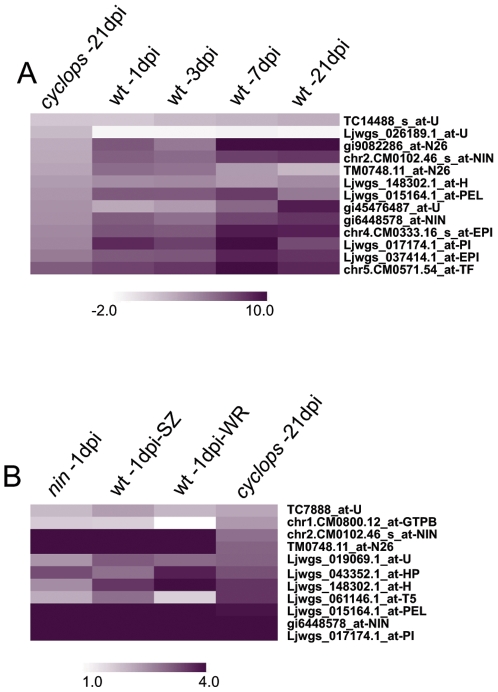
*M. loti* regulated genes in *cyclops* mutant. A) Pattern of the genes regulated by *M. loti* in both *cyclops* and wild-type roots. B) Pattern of genes regulated by *M. loti* in *cyclops* whole roots and in the SZ of *nin* or wild-type. All comparisons were made between *M. loti* treated and uninoculated whole roots (wild-type in panel A and *cyclops*) or SZ (wild-type and *nin* in panel B), respectively. U-unclassified without homolog; N26-nodulin 26; NIN-nodule inception; TF-transcription factor; H-hydrolase; PEL-pectate lyase; EPI-endopeptidase inhibitor; PI-pectinesterase inhibitor; GTPB-GTP binding; T5-taxadienol acetyltransferase; HP-hypothetical protein.

*Cyclops* stands out amongst *Lotus* mutants that develop uninfected nodule primordia. In this class of mutants, *cyclops* is the only one reported to be impaired in mycorrhizal colonization suggesting a more diverse role for the *Cyclops* gene. It has been discussed whether *Cyclops*, in addition to its role in mycorrhization and infection thread formation, is also involved in nodule organogenic pathway progression [7], [15]. In order to have a better understanding of the processes (de)regulated in *cyclops* mutants, we profiled the whole root transcriptome at 21 dpi and compared it to uninoculated mutant root. A direct comparison of *cyclops* and wild-type at 21 dpi revealed large transcriptional differences between these genotypes (44 common IDs, out of which 19 were regulated in opposite directions in the two genotypes). Knowing that *cyclops* nodules at 21 dpi are delayed in development, and in order to have a better understanding of the processes (de)regulated in this mutant, we undertook an unconventional comparison of temporally different developmental stages. The 21 dpi *cyclops* roots were compared to wild-type 1, 3, 7 dpi roots aiming to identify a particular stage of symbiosis which matched the transcript profile of *cyclops* at 21 dpi (Folder S4). This analysis revealed that none of the wild-type transcript profiles from the three stages of symbiosis matched the profile of *cyclops* at 21 dpi. A small number of common IDs (Figure 3A) regulated in a similar fashion were identified in all comparisons (31 with wt 3 dpi, 28 with wt 7 dpi, 25 with wt 21 dpi and 16 with wt 1 dpi- Folder S4). The majority of regulated genes (163 out of 250 IDs) was specific for *cyclops* and were not found regulated in the wild-type inoculated roots at any time point analyzed.

Our analysis therefore showed that genetics and transcriptomics of *cyclops* converge; more genes are regulated by *M*. *loti* in *nin* than in *cyclops* when compared to wild-type. However, we identified *Nin*, *N26*, a *GTP*-binding protein, and a couple of enzymes involved in cell wall loosening (Figure 3B) among the genes (10 IDs), which were regulated by *M. loti* in both mutants and wild-type. In the absence of CYCLOPS, signalling through CCaMK may be partly uncoupled leading to restricted gene regulation. However, among the induced genes we identified *Nin*, which has been shown to be required for cortical cell divisions and nodule primordium formation [48]. These results emphasize the role of CYCLOPS as a central coordinator of endosymbiosis, possibly through its interaction with CCaMK [15], and give the possibility for further in-depth analyses aiming to identify key components controlling infection and/or cortical cell division.

### Impairment in nitrogen fixation leads to a senescent status at the transcript level

In order to understand how transcript regulation and nitrogen fixation relate to nodule development in the later stages, we analyzed the transcriptome of *sen1* and *sst1* mutants. The *sen1* (*sym11*) mutants are arrested just before the onset of nitrogen fixation, they form white nodules with no measurable nitrogen fixation activity and they are nitrogen-starved [57]. The *sst1* (*sym13*) mutant plants develop small inefficient pink nodules that senescence prematurely. Nitrogen fixation is reduced up to 90% compared to wild-type resulting in plants with a nitrogen-deficient phenotype. The *sst1* plants are mutated in a sulfate transporter gene which in wild-type is highly up-regulated between 7 and 14 dpi [37].

Firstly, the transcript profile of 14 and 21 dpi wild-type and 21 dpi mutant nodules was compared to the corresponding profile of uninoculated roots, and only genes, which satisfied the criteria (M, FDR) in these comparisons were further analyzed and presented below.

**Figure 4:**
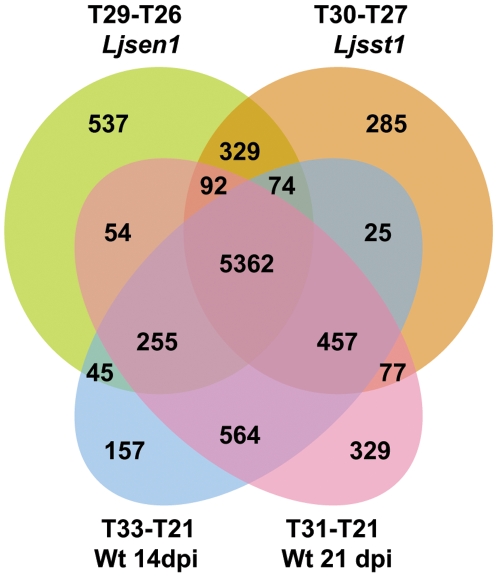
Venn diagram of regulated IDs in *sen1*, *sst1* 21 dpi nodules and wild-type 14 and 21 dpi nodules.

This analysis revealed an impressive number of genes (approximately 8000 IDs) that were differentially expressed in 21 days old nodules of the two mutants with a total of 6362 overlapping IDs, which indicates that many similar processes are initiated in the two genotypes. Out of these, more than 5000 were also found in the 14 and 21 dpi wild-type nodules. This large number of regulated genes shows that most of the cellular processes of wild-type nodules at 14 and 21 dpi are similarly regulated in the two mutants. The *sen1* shared a larger number of regulated genes with wild-type 14 dpi nodules, than *sst1*. However, both mutants had a larger number of regulated genes in common with the wild-type nodules at 21 dpi, showing that nodulation of both mutants was arrested at a developmental stage closer to 21 days than to 14 days (Figure 4).

By contrast, a smaller number of genes (329 IDs) were identified to be both *sen1* and *sst1* specific (not detected in wild-type 14 or 21 dpi nodules). Annotation of these genes includes a large number of enzymes involved in degradation of proteins, lipids, cell wall and carbohydrates such as cysteine endopeptidases, aspartylproteases, serine carboxypeptidases, triacyl glycerol lipases, pectinases and glycosidases. Senescence and cell death related genes were also up-regulated including those encoding for Rhodanese and SPL11 proteins [58], [59]. Numerous transporters belonging to several different categories including peptide, phosphate, and carbohydrate transporters were regulated in both mutants suggesting translocation of compounds from the degrading nodule to the rest of the plant. This might be an indication of a very similar physiological status of the two mutant nodules, which is in good agreement with the early senescent phenotype observed at 21 dpi, a process that in wild-type nodules is normally initiated several weeks later (Reviewed in [60]).

A detailed view of the *sen1* transcriptome identified several genes (537 IDs), which were specifically regulated in this genotype. Genes encoding enzymes involved in starch and sucrose metabolism were frequently observed, which is in agreement with the known accumulation of starch granules in these early senescing nodules (15 out of 98 IDs involved in metabolism). Several transporters (23 IDs) were regulated in *sen1* (18 out of 23 IDs were up-regulated), among them amino acid and peptide transporters that are known to be regulated in response to a variety of environmental and developmental signals (Reviewed in [61]). Previously, it was shown that nitrogen status of plants regulates the expression of amino acid transporters [62] indicating that induction of amino acid transporter genes in *sen1* could be connected to the nitrogen-deficient phenotype and the observed early senescence, which involves nodule protein degradation and translocation of amino acids and peptides to the rest of the plant for recycling.

In a second analysis, the transcript profile of 21 dpi *sen1* and *sst1* nodules was compared to the corresponding profile of 21 dpi wild-type nodules in order to identify genes important for bacteriod differentiation and establishment of symbiotic nitrogen fixation.

Our whole genome analysis identified a large set of genes (987 IDs) specifically regulated in the *sen1* mutant nodules and confirms the majority of the previous observations by Suganuma and co-workers, who identified a total of 93 genes differentially regulated in this mutant compared to wild-type using a cDNA macroarray based on approximately 18,000 nonredundant clones [29]. Among these, our analysis shows that the largest difference compared to wild-type and *sst1* nodule is the nodulin gene *N21*. The function of *N21* is unknown, but it is induced around 7 dpi in wild-type. The white appearance of the *sen1* mutant nodules indicate lack of leghemoglobin, or at least very low levels of the protein. Analysis of leghemoglobin transcript levels in both mutant nodules revealed a level comparable to wild-type, indicating that the reduced level of leghemoglobin protein detected by Western Blot in both mutants is due to translational or post-translational regulation rather than repression of gene expression [37], [57].

A smaller number of genes (254 IDs) were specifically altered in their expression in the *sst1* mutant nodule when compared to wild-type nodule. These included several zinc transporters that were repressed in the mutant. The involvement of zinc transporters during the establishment of symbiotic nitrogen fixation was shown by the identification of a zinc transporter localized in the peribacteriod membrane of soybean [63]. A tight connection between zinc and phosphorous uptake in plants has been observed, and a large requirement for phosphorous was demonstrated both during nodule development and establishment of nitrogen fixation under low nitrogen conditions [64].

The full data set for the *sen1* and *sst1* mutants is available from Folder S5, and profiles of individual genes can be extracted using the publicly available database.

### Gene expression and organ identity

The mature nodule, root, leaf, stem and flower represent the five major organs included in our dataset. To explore the unique expression signatures of these organs, we identified marker genes that were expressed in each of these organs, but not in any of the others. Following the approach of Schmid et al. [65], we used a gcRMA expression value≥6 as a criterion for presence, in combination with a gcRMA expression value≤4 as a criterion for absence. A total of 770 markers were identified using this approach, and of these, 37 were found in nodules, 115 in roots, 116 in leaves, 37 in stems and 465 in flowers (Table S1)

The fact that relatively few genes were found to be nodule markers implies that most genes functioning in the mature nodule are also expressed elsewhere in the plant. 35% of the nodule-specific markers (13 IDs) were assigned to one or more of the four top-level KEGG bins (Figure S4 and Table S2), with a total of 18 assignments. The remaining 65% were unassigned with a homolog (11 IDs) or without a homolog at all (13 IDs). 40% of the nodule-specific markers (15 IDs) (Table S1) were found to be induced late in development, at 21 dpi, and only one was found to be induced earlier, at 7 dpi. Among the nodule markers, we identified transcripts showing homology to RNA-directed DNA polymerases (Ljwgs_129728.2 and TM0459.16), a DNA topoisomerase (Ljwgs_018687.1), a H+-transporting ATPase (Ljwgs_020539.2 and Ljwgs_022205.1), the AP2-domain containing TINY TF (Ljwgs_028899.1), and defense related genes (TM0533.24.1 and chr2.CM0020.37). No previously described nodulins appear to be expressed exclusively in nodules (Folder S6). In all organs, less than half of the marker genes were classified within KEGG, however the nodule was the organ with the largest fraction of genes showing no homology at all. At the other extreme was the flower and the stem with only 8% of markers (39 of 465 in the flower and 3 of 37 in the stem) showing no homology to annotated genes. The most prominent category of the KEGG bins was metabolism, which was especially pronounced in the root, where more than 80% of the categorized marker genes (39 out of 46) were assigned to this bin.

To investigate whether the organ marker gene products are associated with specific biological processes, cellular components or molecular functions, we summarized GO annotation available for 16 nodule, 68 root, 75 leaf, 28 stem and 329 flower markers (Table 2). In the nodule, 4 of the 16 markers are involved in responses to biotic or abiotic stimuli, and, in the root, 14 out of 68 markers are oxidoreductases. In both root and stem, a large fraction of markers are transcriptional regulators. Several categories are statistically over-represented in the leaf and flower. Most notably, several markers seem to be membrane associated in both organs. Also, 85 of 329 flower markers with a GO representation are hydrolases.

**Table 2:** Organ marker genes assigned to GOslim categories from the GOA database at EBI.

			Nodule		Root		Leaf		Stem		Flower	
biological process	GO term	**All**	n	p	n	p	n	p	n	p	n	p
	biosynthetic process	80					1	0.20			1	0.63
	Cell differentiation	62									1	0.53
	cellular process	3017	3	0.26	11	0.13	12	0.13	5	0.20	30	0.90
	electron transport	278					**4**	**0.01**				
	macromolecule metabolic process	5844	1	0.98	3	1.00	5	1.00	2	0.99	36	1.00
	metabolic process	634			3	0.21	**5**	**0.03**			12	0.09
	multicellular organismal development	304			1	0.54			1	0.27	**8**	**0.03**
	regulation of biological process	2308	1	0.76	10	0.06	4	0.89	**6**	**0.03**	22	0.91
	response to stimulus	2148	**4**	**0.03**	9	0.09	8	0.25	3	0.39	7	1.00
	secretion	83			1	0.19					3	0.08
	transport	1080	1	0.48	5	0.14	6	0.08	1	0.68	**26**	**0.00**
cellular component	cell	587					1	0.81			6	0.72
	chromosome	106	1	0.06								
	cytoplasm	2574	2	0.46	1	1.00	5	0.85	3	0.51	18	1.00
	external encapsulating structure	127			**3**	**0.00**			1	0.12	**14**	**0.00**
	extracellular region	23			1	0.06					**2**	0.03
	membrane	2383	2	0.42	4	0.86	**14**	**0.01**	2	0.72	**39**	**0.04**
	nucleus	1493	1	0.60	1	0.98			3	0.20	3	1.00
molecular function	binding	7710	2	0.97	15	0.91	18	0.84	4	0.98	75	0.99
	catalytic activity	1538					4	0.63			4	1.00
	channel activity	171			1	0.35	1	0.38			5	0.06
	electron transporter activity	87					**2**	**0.02**				
	enzyme regulator activity	311			1	0.55			1	0.28	**11**	**0.00**
	hydrolase activity	3758	4	0.17	12	0.23	9	0.73	3	0.77	**85**	**0.00**
	ion transmembrane transporter activity	604	1	0.30	1	0.79	**6**	**0.01**	2	0.13	**13**	**0.04**
	isomerase activity	246									1	0.95
	Ligase activity	518					2	0.42			1	1.00
	lyase activity	426			2	0.29	2	0.33			9	0.08
	motor activity	176	1	0.10							3	0.36
	oxidoreductase activity	1299			**14**	**0.00**	7	0.07	3	0.15	16	0.52
	protein binding	1658			3	0.80	5	0.49	1	0.83	20	0.55
	signal transducer activity	100					**2**	**0.03**				
	structural molecule activity	455					1	0.72			1	1.00
	transcription regulator activity	2106	1	0.73	**11**	**0.02**	6	0.54	**8**	**0.00**	29	0.27
	transferase activity	5437	3	0.65	8	0.98	8	0.99	5	0.69	62	0.74
	translation regulator activity	183									2	0.66
	transporter activity	978					**8**	**0.01**	1	0.64	15	0.22

GOslim categories are grouped by major Gene Ontology group. For each organ, the number of genes (n) assigned to each category is indicated along with a probability (p) that the intersection between organ markers and GOslim category occurs by chance. The total number of sequences on the chip mapped to each category is shown after each category name (All). Significant intersections are in boldface (p<0.05).

Looking at lower level classifications, four major groups can be distinguished within the markers, namely transcription factors, kinases, transporters and defense-related genes. Figure S5 shows a heat map of 179 markers distributed between these four major categories found by a keyword search in the Gene Ontology description of the best BLASTX hit of all markers (Table S2).

In addition to the organ marker genes, we identified a multitude of genes (6075 IDs) that were expressed at comparable levels (with a minimum expression of 6, and a range<1) in all five organs (Table S1). Interestingly, several genes show remarkably little variation across all tissues, treatments and genotypes (Table S3). Among these, we identified one gene (gi45348456) showing homology to *Arabidopsis* ubiquitin (UBQ9, AT5G37640.1) and another one (Ljwgs_018207.1) showing homology to a regulatory subunit of Protein Phosphatase 2A (PDF1, AT5G37640.7), which has been suggested as a superior reference gene for normalization of gene transcript levels in *Arabidopsis* [66]. This list can serve as a starting point for testing new reference genes in *Lotus*.

### *Lotus* acclimation to nitrate leads to major changes in the shoot transcript profile

**Figure 5:**
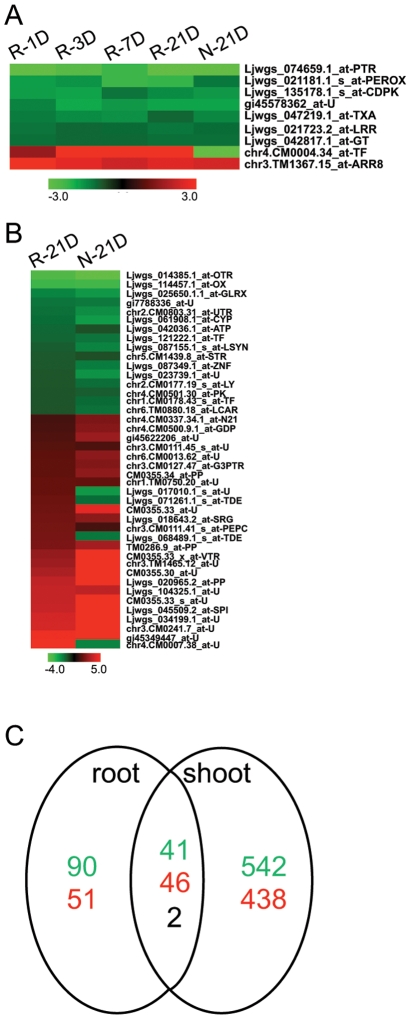
Transcript regulation by nitrate in *Lotus.* A) The pattern of IDs found to be regulated by nitrate and *M. loti* inoculation (1 dpi to 21 dpi). B) The pattern of IDs regulated by nitrate and *M. loti* inoculation (21 dpi). C) Number of IDs down-regulated (green), up-regulated (red) or differently regulated (black) by nitrate in *Lotus* roots and shoots when compared to untreated corresponding tissues. (R-*M. loti*, N-nitrate) PTR-peptide transporter; PEROX-peroxidase; CDPK-calcium and calmodulin kinase; U-unknown; TXA-TXA synthase; LRR-leucine rich repeat protein; GT-UDP- glycosyltransferase; TF-transcription factor; ARR8-response regulator 8; OTR-ornithine transporter; OX-oxidoreductase; GLRX-glutaredoxin; UTR-urea transporter; CYP-Cytochrome P450; ATP-ATPase; LSYN-lupeol synthase; STR-sugar transporter; ZNF-zinc finger; LY-lyase; PK-protein kinase; LCAR-L-carbamoylase; N21-nodulin 21; GDP-glycerophosphoryl diester phosphodiesterase; G3PTR-G-3-P transporter; PP-phosphatase; TDE-tumor differently expressed; SRG-senescence related gene; PEPC-phosphoenolpyruvate carboxylase; VTR-vacuolar transporter; SPI-serine protease inhibitor).

Compared to the rhizobia-inoculated roots, acclimation to nitrate as nitrogen source leads to regulation of a limited number of genes in *Lotus*. Genes corresponding to only 230 IDs were found differently regulated in the nitrate-grown roots compared to untreated roots, and out of these only 90 were specific for the nitrate treatment and were not found in the pool of genes regulated in response to *M. loti*. These included homologs of nitrate, peptide- and phosphate-transporters that were previously found regulated in *Arabidopsis* in response to inorganic nitrogen (reviewed in [67]). Interestingly, nine of the genes that were regulated by inoculation with *M. loti* were regulated similarly in the nitrate grown roots. Among these, genes encoding an UDP-glycosyltransferase and ARR8 are found, indicating that cytokinin signalling may be important in both symbiotic and inorganic nitrogen plant nutrition (Figure 5A and Folder S7). Not surprisingly, nitrate-grown roots have more differentially expressed genes in common with the 21 dpi roots, compared to the other analyzed time points. These included genes for anthocyanidin synthase and a urea transporter, which were down-regulated, and a couple of transcriptional regulators and *N21*, which were up-regulated (Figure 5B).

*Lotus* plants grown for 3 weeks on nitrate, displayed the most dramatic change in the transcriptome of the shoot, compared to root. Genes corresponding to more than a thousand IDs were differently regulated in the aerial part of the plant by this growth condition, and among these, 90 were found affected in both roots and shoots (Figure 5C and Folder S7).

## Materials and Methods

### Plant material and experimental setup

*Lotus japonicus* (ecotype Gifu) and eight mutant plants (*nfr1–2*, *nfr5–2*, *nup133–3*, *nin-2*, *cyclops (sym 6–2)*, *sen1*, *sst1*, *har1–3*) were used for our analyses. All mutants were obtained upon tissue culture of Gifu plants using a *Ac/Ds* tagging element [68]. However, only *nin-1* mutant plants were *Ac* tagged [20]. The genetic variability in the *Lotus* tissue culture mutant collection is low. Retroelements with only two-fold copy number increase constitute a main cause of mutation in this material [69], [70]. Nevertheless, mutant plants from the first backcross generation of *nfr1–1* and *cyclops* were used as they were available. Tissues were primarily sampled from three-week old seedlings, and the symbiotic response was profiled across several genotypes and time points spanning the developmental process from early signalling to the onset of nitrogen fixation in mature nodules. All plants were grown under the same conditions and all samples from a particular condition/treatment were harvested at the same time of day. However, not all environmental factors, such as greenhouse light conditions and temperature fluctuations, are easily controlled. Therefore, we sought to randomize such influences by performing most experiments in triplicates from three spatially and temporally separated batches of plants, with all conditions represented in each batch.

### Plant growth conditions and treatment

Seeds were prepared for germination by scarifying seed coats in concentrated sulfuric acid for 5 minutes and then sterilized by submersion in 10×diluted hypochlorite for approximately 12 minutes. Plants were grown in washed and sterilized Leca® (grain size 4–10 mm) and supplied with half-strength B&D medium [71]. Plants on full nutrition were supplied with 5 mM NO^3−^. Nodulating and Nod factor treated plants were supplied with 1 mM NO^3−^. The photoperiod was 16 h light and 8 h dark. Inoculation with the *Lotus japonicus* symbiont, *Mesorhizobium loti* strain R7A, was performed by growing the bacterial culture to an OD_600_ of 0.211 and diluting it 100 times in half-strength B&D. Nod factor was isolated from the same rhizobial strain [72] and diluted to a final concentration of 10^−7^ M. For the 24 h Nod factor treatment, 21 days old Leca-grown plants were transferred to a new container with 100 ml of Nod factor liquid solution to ensure a full immersion of roots. At harvest, tissues were snap-frozen in liquid nitrogen and stored at −80°C. Root susceptible zones and nodules were excised immediately and stored in Eppendorf tubes.

### RNA extraction

Buffer containing 1% w/v SDS, 2% w/v polyvinylpyrrolidone (PVP), 10 mM DTT and 100 mM β-mercaptoethanol was added to samples already homogenized in liquid nitrogen, followed by addition of borate-buffer (20 mM Na-Borate, 30 mM EGTA, 5 mM EDTA, 1% w/v Na-Deoxycholate) pre-heated to 95°C. Nucleic acids were extracted with chloroform:isoamylalcohol (24∶1) and precipitated with isopropanol. After re-suspension in DEPC-treated H_2_O, RNA was precipitated in 2M LiCl overnight, washed twice with cold 70% ethanol, dried and re-suspended in DEPC H_2_O. Finally, all samples were DNase treated.

### Target preparation

First and second strand cDNA synthesis and Biotin labelling was performed using the Affymetrix® One-Cycle cDNA Synthesis Kit and the GeneChip IVT Labeling Kit, according to the standard protocol “Eukaryotic Sample and Array Processing”, which can be obtained from http://www.affymetrix.com/. 3 µg of total RNA was used for each reaction.

### GeneChip hybridization

One target sample was prepared for each biological replica and hybridized to a single *Lotus* GeneChip®. The German Science Centre for Genome Research carried out all hybridizations (RZPD) in Berlin, Germany (http://www.rzpd.de).

### Data analysis

Pre-analysis data quality assessment was done by visual inspection of individual false colour hybridization images and standard diagnostic plots of probe level intensity distributions using BioConductor (http://www.bioconductor.org/) and R software. All data were analyzed using the BioConductor software project and the statistical language R. Raw data from all hybridizations were background corrected, normalized and summarized using gcRMA [73] as implemented in R with default parameters [74]. Consequently, all data are log2 transformed. Before statistical analysis, bacterial genes were filtered out along with genes called absent by the mas5calls() function. After filtering, a total of 44.040 probe sets were included in further analyses. Significant genes were identified using the Limma package [75]. Unless otherwise stated, an FDR (False Discovery Rate) corrected p-value≤0.05 was used as the criterion for significance, in combination with a |M|≥1 filter, where M is the log2 ratio of average expression values from any two conditions.

### Database creation and data availability

Expression data was organized in a database holding raw and normalized expression data together with information about sample generation and characteristics. The database is organized as a web-accessible resource that can be mined by directing a web browser to http://www.brics.dk/cgi-compbio/Niels/index.cgi. The website provides a visualization tool that can display the expression levels of one or several genes of interest across all conditions. Genes that behave similarly to a query gene across conditions can be identified using the *Lotus* Profile Matching tool. This tool calculates the distance between (Euclidian) or covariance of (Pearson) expression vectors, but instead of clustering and producing graphical output, it simply returns a user-defined number of closest matches based on the similarity measure chosen (Euclidian or Pearson). For convenience, the results can be copied to the visualization query window for quick and easy inspection. There is also an option to export expression data for matching genes.

Furthermore, all gene expression data have been deposited in the ArrayExpress database (http://www.ebi.ac.uk/microarray-as/ae/) under the accession number E-TABM-715.

### Properties of the dataset and the analysis

The number of detected probe sets was similar in most samples, ranging from 36.2% to 51.6% (average 47.9%). These levels are slightly lower than those previously reported in an *Arabidopsis* transcript profiling study (55% to 61% detected genes) [65], and may reflect a larger number of false gene predictions (pseudogenes) from the genome annotation. When ascribing putative gene function to features on the *Lotus japonicus* GeneChip®, we relied on sequence homology to existing database entries. More specifically, a bioinformatics system was created to assign sequences to GeneBins modelled on the Kyoto Encyclopedia of Genes and Genomes (KEGG) classification and to retrieve pathway information [76].

For encapsulating organ marker gene functions we have turned to a subset of Gene Ontology (GO) categories (a GOslim). The GO slim is essentially a cut-down version of the GO system and was designed to summarize large sets of GO annotation data. Organ marker genes were compared to the composition of GOslim categories provided by the GOA database at EBI and tested for statistical over-representation using the hyper-geometric distribution. The computed p-values represent the probability that the intersection of organ marker genes with the list of genes belonging to the relevant GOslim category occurs by chance.

### Various websites used for data analysis

http://www.brics.dk/cgi-compbio/Niels/index.cgi: contains the *L. japonicus* publicly available database holding raw and normalized expression data together with information about sample generation and characteristics.

http://www.ebi.ac.uk/GOA/: GOA database at EBI.

http://bioinfoserver.rsbs.anu.edu.au/utils/PathExpress-lotus/: and http://bioinfoserver.rsbs.anu.edu.au/utils/GeneBins-lotus/: databases that currently support the functional classification of expression data from *L. japonicus* 52K.

www.r-project.org : R- software environment for statistical computing and graphics.

http://www.bioconductor.org/: open source and open development software project for the analysis and comprehension of genomic data.

## Supporting Information

Figure S1 Tissue-, genotype- and treatment effects on the *Lotus* transcriptome are sampled in 38 conditions.
(2.52 MB EPS)

Figure S2 Number of probe sets regulated in *L. japonicus* roots upon inoculation with *M. loti* at 1, 3, 7 and 21 dpi. Each colour represents a particular comparison.
(0.28 MB EPS)

Figure S3 *Lotus* root responses to *M. loti* inoculation at 1 dpi.Pattern of the predicted TF (A, B and C), receptors and kinases (D, E and F) regulated by *M. loti* in the SZ (A and D), the whole root (B and E) and in the SZ by both *M. loti* and Nod factors (NF) (C and F). Pattern of the IDs involved in auxin and ethylene (G) and cytokinin (H) signalling regulated by *M. loti* or *M. loti* Nod factors in the SZ or the whole root (R). The corresponding FDR values are shown in the tables to the left.
(1.70 MB EPS)

Figure S4 KEGG assignment of organ specific IDs. Blue: number of probe sets classified, Red: probe sets with a homologue, but without a KEGG bin assignment, Green: probe sets with no homolog. Bar plots show the number of assignments in each of the four major KEGG bins: 1: Metabolism; 2: Genetic Information Processing; 3: Environmental Information Processing; 4: Cellular Processes.
(1.41 MB EPS)

Figure S5 Organ markers grouped by functional category.
(1.06 MB EPS)

Table S1 L. japonicus organ markers
(1.52 MB XLS)

Table S2 KEGG assignment and functional categories by organ
(0.08 MB XLS)

Table S3 L. japonicus constitutively expressed genes
(0.08 MB XLS)

Folder S1 Inoculation time series
(2.84 MB ZIP)

Folder S2 Inoculation 1 day
(2.85 MB ZIP)

Folder S3 Nod-minus mutants
(0.70 MB ZIP)

Folder S4 Cyclops mutant
(0.59 MB ZIP)

Folder S5 Fix-minus mutants
(9.19 MB ZIP)

Folder S6 Organs
(0.23 MB ZIP)

Folder S7 Nitrate
(0.85 MB ZIP)

## References

[1] Tirichine L, Imaizumi-Anraku H, Yoshida S, Murakami Y, Madsen LH (2006). Deregulation of a Ca2+/calmodulin-dependent kinase leads to spontaneous nodule development.. Nature.

[2] Tirichine L, James EK, Sandal N, Stougaard J (2006). Spontaneous root-nodule formation in the model legume *Lotus japonicus*: A novel class of mutants nodulates in the absence of rhizobia.. Mol Plant Microbe Interact.

[3] Oldroyd GE, Downie JA (2004). Calcium, kinases and nodulation signalling in legumes.. Nat Rev Mol Cell Biol.

[4] Madsen EB, Madsen LH, Radutoiu S, Olbryt M, Rakwalska M (2003). A receptor kinase gene of the LysM type is involved in legume perception of rhizobial signals.. Nature.

[5] Radutoiu S, Madsen LH, Madsen EB, Felle HH, Umehara Y (2003). Plant recognition of symbiotic bacteria requires two LysM receptor-like kinases.. Nature.

[6] Radutoiu S, Madsen LH, Madsen EB, Jurkiewicz A, Fukai E (2007). LysM domains mediate lipochitin-oligosaccharide recognition and nfr genes extend the symbiotic host range.. EMBO J.

[7] Kistner C, Winzer T, Pitzschke A, Mulder L, Sato S (2005). Seven *Lotus japonicus* genes required for transcriptional reprogramming of the root during fungal and bacterial symbiosis.. Plant Cell.

[8] Stracke S, Kistner C, Yoshida S, Mulder L, Sato S (2002). A plant receptor-like kinase required for both bacterial and fungal symbiosis.. Nature.

[9] Endre G, Kereszt A, Kevei Z, Mihacea S, Kalo P (2002). A receptor kinase gene regulating symbiotic nodule development.. Nature.

[10] Imaizumi-Anraku H, Takeda N, Charpentier M, Perry J, Miwa H (2004). Plastid proteins crucial for symbiotic fungal and bacterial entry into plant roots.. Nature.

[11] Ane JM, Kiss GB, Riely BK, Penmetsa RV, Oldroyd GE (2004). *Medicago truncatula* DMI1 required for bacterial and fungal symbioses in legumes.. Science.

[12] Kanamori N, Madsen LH, Radutoiu S, Frantescu M, Quistgaard EM (2006). A nucleoporin is required for induction of Ca2+ spiking in legume nodule development and essential for rhizobial and fungal symbiosis.. Proc Natl Acad Sci U S A.

[13] Saito K, Yoshikawa M, Yano K, Miwa H, Uchida H (2007). NUCLEOPORIN85 is required for calcium spiking, fungal and bacterial symbioses, and seed production in *Lotus japonicus*.. Plant Cell.

[14] Miwa H, Sun J, Oldroyd GE, Downie JA (2006). Analysis of nod-factor-induced calcium signaling in root hairs of symbiotically defective mutants of *Lotus japonicus*.. Mol Plant Microbe Interact.

[15] Yano K, Yoshida S, Müller J, Singh S, Banba M, Vickers K, Markmann K, White C, Schuller B, Sato S, Asamizu E, Tabata S, Murooka Y, Perry J, Wang TL, Kawaguchi M, Imaizumi-Anraku H, Hayashi M, Parniske M (2008). CYCLOPS, a mediator of symbiotic intracellular accommodation.. Proc Natl Acad Sci U S A.

[16] Chen C, Ane JM, Zhu H (2008). OsIPD3, an ortholog of the *Medicago truncatula* DMI3 interacting protein IPD3, is required for mycorrhizal symbiosis in rice.. New Phytol.

[17] Messinese E, Mun JH, Yeun LH, Jayaraman D, Rouge P (2007). A novel nuclear protein interacts with the symbiotic DMI3 calcium- and calmodulin-dependent protein kinase of *Medicago truncatula*.. Mol Plant Microbe Interact.

[18] Lévy J, Bres C, Geurts R, Chalhoub B, Kulikova O, Duc G, Journet EP, Ané JM, Lauber E, Bisseling T, Dénarié J, Rosenberg C, Debellé F (2004). A putative Ca2+ and calmodulin-dependent protein kinase required for bacterial and fungal symbioses.. Science.

[19] Smit P, Raedts J, Portyanko V, Debellé F, Gough C, Bisseling T, Geurts R (2005). NSP1 of the GRAS protein family is essential for rhizobial Nod factor-induced transcription.. Science.

[20] Schauser L, Roussis A, Stiller J, Stougaard J (1999). A plant regulator controlling development of symbiotic root nodules.. Nature.

[21] Heckmann AB, Lombardo F, Miwa H, Perry JA, Bunnewell S (2006). *Lotus japonicus* nodulation requires two GRAS domain regulators, one of which is functionally conserved in a non-legume.. Plant Physiol.

[22] Kalo P, Gleason C, Edwards A, Marsh J, Mitra RM (2005). Nodulation signaling in legumes requires NSP2, a member of the GRAS family of transcriptional regulators.. Science.

[23] Marsh JF, Rakocevic A, Mitra RM, Brocard L, Sun J (2007). *Medicago truncatula* NIN is essential for rhizobial-independent nodule organogenesis induced by autoactive calcium/calmodulin-dependent protein kinase.. Plant Physiol.

[24] El Yahyaoui F, Kuster H, Amor BB, Hohnjec N, Puhler A (2004). Expression profiling in *Medicago truncatula* identifies more than 750 genes differentially expressed during nodulation, including many potential regulators of the symbiotic program.. Plant Physiol.

[25] Lohar DP, Sharopova N, Endre G, Penuela S, Samac D (2006). Transcript analysis of early nodulation events in *Medicago truncatula*.. Plant Physiol.

[26] Benedito VA, Torres-Jerez I, Murray JD, Andriankaja A, Allen S (2008). A gene expression atlas of the model legume *Medicago truncatula*.. Plant J.

[27] Mitra RM, Shaw SL, Long SR (2004). Six nonnodulating plant mutants defective for Nod factor-induced transcriptional changes associated with the legume-rhizobia symbiosis.. Proc Natl Acad Sci U S A.

[28] Kouchi H (2004). Large-scale analysis of gene-expression profiles during early stages of root nodule formation in a model legume, *Lotus japonicus*.. DNA Research.

[29] Suganuma N, Yamamoto A, Itou A, Hakoyama T, Banba M (2004). cDNA macroarray analysis of gene expression in ineffective nodules induced on the *Lotus japonicus sen1* mutant.. Mol Plant Microbe Interact.

[30] Colebatch G, Desbrosses G, Ott T, Krusell L, Montanari O (2004). Global changes in transcription orchestrate metabolic differentiation during symbiotic nitrogen fixation in *Lotus japonicus*.. Plant J.

[31] Deguchi Y, Banba M, Shimoda Y, Chechetka SA, Suzuri R (2007). Transcriptome profiling of *Lotus japonicus* roots during arbuscular mycorrhiza development and comparison with that of nodulation.. DNA Res.

[32] Sato S, Nakamura Y, Kaneko T, Asamizu E, Kato T (2008). Genome structure of the legume, *Lotus japonicus*.. DNA Res.

[33] Krusell L, Madsen LH, Sato S, Aubert G, Genua A (2002). Shoot control of root development and nodulation is mediated by a receptor-like kinase.. Nature.

[34] Sampedro J, Cosgrove DJ (2005). The expansin superfamily.. Genome Biol.

[35] Fudali S, Janakowski S, Sobczak M, Griesser M, Grundler FM (2008). Two tomato alpha-expansins show distinct spatial and temporal expression patterns during development of nematode-induced syncytia.. Physiol Plant.

[36] Wieczorek K, Hofmann J, Blochl A, Szakasits D, Bohlmann H (2008). *Arabidopsis* endo-1,4-beta-glucanases are involved in the formation of root syncytia induced by *Heterodera schachtii*.. Plant J.

[37] Krusell L, Krause K, Ott T, Desbrosses G, Kramer U (2005). The sulfate transporter SST1 is crucial for symbiotic nitrogen fixation in *Lotus japonicus* root nodules.. Plant Cell.

[38] Cohn JR, Uhm T, Ramu S, Nam YW, Kim DJ (2001). Differential regulation of a family of apyrase genes from *Medicago truncatula*.. Plant Physiol.

[39] Navarro-Gochicoa MT, Camut S, Niebel A, Cullimore JV (2003). Expression of the apyrase-like *APY1* genes in roots of *Medicago truncatula* is induced rapidly and transiently by stress and not by *Sinorhizobium meliloti* or nod factors.. Plant Physiol.

[40] Kim SY, Sivaguru M, Stacey G (2006). Extracellular ATP in plants. Visualization, localization, and analysis of physiological significance in growth and signaling.. Plant Physiol.

[41] Govindarajulu M, Kim SY, Libault M, Berg RH, Tanaka K, Stacey G, Taylor CG (2008). GS52 ecto-apyrase plays a critical role during soybean nodulation.. Plant Physiol.

[42] Culley DE, Horovitz D, Hadwiger LA (1995). Molecular characterization of disease-resistance response gene *DRR206-d* from *Pisum sativum* (L.).. Plant Physiol.

[43] Whitham S, Dinesh-Kumar SP, Choi D, Hehl R, Corr C (1994). The product of the tobacco mosaic virus resistance gene *N*: Similarity to toll and the interleukin-1 receptor.. Cell.

[44] Vercammen D, Belenghi B, van de Cotte B, Beunens T, Gavigan JA (2006). Serpin1 of *Arabidopsis thaliana* is a suicide inhibitor for metacaspase 9.. J Mol Biol.

[45] Nukui N, Ezura H, Yuhashi K, Yasuta T, Minamisawa K (2000). Effects of ethylene precursor and inhibitors for ethylene biosynthesis and perception on nodulation in *Lotus japonicus* and *Macroptilium atropurpureum*.. Plant Cell Physiol.

[46] Oldroyd GE, Engstrom EM, Long SR (2001). Ethylene inhibits the nod factor signal transduction pathway of *Medicago truncatula*.. Plant Cell.

[47] Murray JD, Karas BJ, Sato S, Tabata S, Amyot L (2007). A cytokinin perception mutant colonized by rhizobium in the absence of nodule organogenesis.. Science.

[48] Tirichine L, Sandal N, Madsen LH, Radutoiu S, Albrektsen AS (2007). A gain-of-function mutation in a cytokinin receptor triggers spontaneous root nodule organogenesis.. Science.

[49] Gonzalez-Rizzo S, Crespi M, Frugier F (2006). The *Medicago truncatula* CRE1 cytokinin receptor regulates lateral root development and early symbiotic interaction with *Sinorhizobium meliloti*.. Plant Cell.

[50] D'Agostino IB, Deruere J, Kieber JJ (2000). Characterization of the response of the *Arabidopsis* response regulator gene family to cytokinin.. Plant Physiol.

[51] Oldroyd GE, Downie JA (2008). Coordinating nodule morphogenesis with rhizobial infection in legumes.. Annu Rev Plant Biol.

[52] Remans T, Nacry P, Pervent M, Filleur S, Diatloff E (2006). The *Arabidopsis* NRT1.1 transporter participates in the signaling pathway triggering root colonization of nitrate-rich patches.. Proc Natl Acad Sci U S A.

[53] Dewitte W, Scofield S, Alcasabas AA, Maughan SC, Menges M (2007). *Arabidopsis* CYCD3 D-type cyclins link cell proliferation and endocycles and are rate-limiting for cytokinin responses.. Proc Natl Acad Sci U S A.

[54] Hala M, Cole R, Synek L, Drdova E, Pecenkova T (2008). An exocyst complex functions in plant cell growth in *Arabidopsis* and tobacco.. Plant Cell.

[55] Weis K (2002). Nucleocytoplasmic transport: Cargo trafficking across the border.. Curr Opin Cell Biol.

[56] Menon BB, Sarma NJ, Pasula S, Deminoff SJ, Willis KA (2005). Reverse recruitment: The Nup84 nuclear pore subcomplex mediates Rap1/Gcr1/Gcr2 transcriptional activation.. Proc Natl Acad Sci U S A.

[57] Suganuma N, Nakamura Y, Yamamoto M, Ohta T, Koiwa H (2003). The *Lotus japonicus Sen1* gene controls rhizobial differentiation into nitrogen-fixing bacteroids in nodules.. Mol Genet Genomics.

[58] Caplan JL, Mamillapalli P, Burch-Smith TM, Czymmek K, Dinesh-Kumar SP (2008). Chloroplastic protein NRIP1 mediates innate immune receptor recognition of a viral effector.. Cell.

[59] Yin Z, Chen J, Zeng L, Goh M, Leung H (2000). Characterizing rice lesion mimic mutants and identifying a mutant with broad-spectrum resistance to rice blast and bacterial blight.. Mol Plant Microbe Interact.

[60] Puppo A, Groten K, Bastian F, Carzaniga R, Soussi M (2005). Legume nodule senescence: Roles for redox and hormone signalling in the orchestration of the natural aging process.. New Phytol.

[61] Liu X, Bush DR (2006). Expression and transcriptional regulation of amino acid transporters in plants.. Amino Acids.

[62] Miller AJ, Fan X, Shen Q, Smith SJ (2008). Amino acids and nitrate as signals for the regulation of nitrogen acquisition.. J Exp Bot.

[63] Moreau S, Thomson RM, Kaiser BN, Trevaskis B, Guerinot ML (2002). GmZIP1 encodes a symbiosis-specific zinc transporter in soybean.. J Biol Chem.

[64] Leidi EOEO, Rodriguez- Navarro DN (2000). Nitrogen and phosphorus availability limit N_2_ fixation in bean.. New Phytol.

[65] Schmid M, Davison TS, Henz SR, Pape UJ, Demar M (2005). A gene expression map of *Arabidopsis thaliana* development.. Nat Genet.

[66] Czechowski T, Stitt M, Altmann T, Udvardi MK, Scheible WR (2005). Genome-wide identification and testing of superior reference genes for transcript normalization in *Arabidopsis*.. Plant Physiol.

[67] Forde BG (2002). Local and long-range signaling pathways regulating plant responses to nitrate.. Annu Rev Plant Biol.

[68] Schauser L, Handberg K, Sandal N, Stiller J, Thykjaer T (1998). Symbiotic mutants deficient in nodule establishment identified after T-DNA transformation of *Lotus japonicus*.. Mol Gen Genet.

[69] Madsen LH, Fukai E, Radutoiu S, Yost CK, Sandal N (2005). LORE1, an active low-copy-number TY3-gypsy retrotransposon family in the model legume lotus japonicus.. Plant J.

[70] Fukai E, Dobrowolska AD, Madsen LH, Madsen EB, Umehara Y (2008). Transposition of a 600 thousand-year-old LTR retrotransposon in the model legume lotus japonicus.. Plant Mol Biol.

[71] Broughton D (1971). Media for legumes.. Biochem J.

[72] Lopez-Lara IM, van den Berg JD, Thomas-Oates JE, Glushka J, Lugtenberg BJ (1995). Structural identification of the lipo-chitin oligosaccharide nodulation signals of *Rhizobium loti*.. Mol Microbiol.

[73] Wu Z, Irizarry RA, Gentleman R, Murillo FM, Spencer F (2004). A model based background adjustment for oligonucleotide expression arrays.. Http://www.

[74] Gautier L, Cope L, Bolstad BM, Irizarry RA (2004). Affy–analysis of affymetrix GeneChip data at the probe level.. Bioinformatics.

[75] Smyth GK, Gentleman R, Carey S, Dudoit R, Irizarry R, Huber W (2005). Limma: Linear models for microarray data.. Bioinformatics and Computational Biology Solutions using R and Bioconductor.

[76] Goffard N, Weiller G (2007). GeneBins: A database for classifying gene expression data, with application to plant genome arrays.. BMC Bioinformatics.

