# Supplementary material for: Dissection of Symbiosis and Organ Development by Integrated Transcriptome Analysis of Lotus japonicus Mutant and Wild-Type Plants
Source: PLoS One. 2009 Aug 7;4(8):e6556. doi: 10.1371/journal.pone.0006556 (PMC2717213; doi:10.1371/journal.pone.0006556)
Supplement: Folder S1 — Inoculation time series (2.84 MB ZIP) [file pone.0006556.s009.zip › S1.INOCULATION TIME SERIES copy/1.2.Stage(s) specific transcript analysis/-1.2.Comparisons overview.doc]

| Contrast code | T13 - T21 | T15 - T21 | T34 - T21 | T32 - T21 |
| --- | --- | --- | --- | --- |
| Folder/Contrast | 1dpi/uninoc. | 3dpi/uninoc. | 7dpi/uninoc. | 21dpi/uninoc. |
| 1.2.1 |  |  |  |  |
| 1.2.2 |  |  |  |  |
| 1.2.3 |  |  |  |  |
| 1.2.4 |  |  |  |  |
| 1.2.5 |  |  |  |  |
| 1.2.6 |  |  |  |  |
| 1.2.7 |  |  |  |  |
| 1.2.8 |  |  |  |  |
| 1.2.9 |  |  |  |  |
| 1.2.10 |  |  |  |  |
| 1.2.11 |  |  |  |  |
| 1.2.12 |  |  |  |  |
| 1.2.13 |  |  |  |  |
| 1.2.14 |  |  |  |  |
| 1.2.15 |  |  |  |  |

Each folder (1.2.1. to 1.2.15) contains the analysis of *L. japonicus* IDs regulated by *M. loti* at particular time point(s). The whole root transcript profile of each developmental stage (1dpi, 3dpi, 7dpi, 21dpi) was compared to the profile of uninoculated roots. IDs that satisfied the established M and FDR criteria in the contrasts marked in green but not in those marked in red are listed in the file <-1.1.#. ID list>.
