# Supplementary material for: Dissection of Symbiosis and Organ Development by Integrated Transcriptome Analysis of Lotus japonicus Mutant and Wild-Type Plants
Source: PLoS One. 2009 Aug 7;4(8):e6556. doi: 10.1371/journal.pone.0006556 (PMC2717213; doi:10.1371/journal.pone.0006556)
Supplement: Folder S1 — Inoculation time series (2.84 MB ZIP) [file pone.0006556.s009.zip › S1.INOCULATION TIME SERIES copy/Supporting Folder 1 Info.doc]

Supporting Folder 1. **Inoculation time series**

**Folder 1.1.Stage(s) total transcript analysis**

<-1.1. Comparisons overview> describes the comparisons analysed in folder 1.1.

**Folder 1.1.# – Analysis of *L. japonicus* IDs regulated by *M. loti* at particular time point(s) as mentioned in the file <-1.1. Comparisons overview>**

<-1.1.#.ID list> List of IDs and their corresponding M and FDR values in particular contrast(s) mentioned in the file <-1.1. Comparisons overview>.

<-1.1.#.ID-ANU.txt> putative annotation of the IDs found in the file <-1.1.#.ID list>.

<1.1.#.abscisic acid.txt> IDs identified by performing a “abscisic” keyword search in the <-1.1.#.ID-ANU.txt>.

<1.1.#.auxin.txt> IDs identified by performing a “auxin” keyword search in the <-1.1.#.ID-ANU.txt>.

<1.1.#.brassinosteroid.txt> IDs identified by performing a “brassinosteroid” and “BRASSINOSTEROID” keywords search in the <-1.1.#.ID-ANU.txt>.

<1.1.#.cytokinin.txt> IDs identified by performing a “cytokinin” keyword search in the <-1.1.#.ID-ANU.txt>.

<1.1.#.defense.txt> IDs identified by performing a “defense” keyword search in the <-1.1.#.ID-ANU.txt>.

<1.1.#.ethylene.txt> IDs identified by performing a “ethylene” keyword search in the <-1.1.#.ID-ANU.txt>.

<1.1.#.gibberellin.txt> IDs identified by performing a “gibberellin” keyword search in the <-1.1.#.ID-ANU.txt>.

<1.1.#.jasmonic acid.txt> IDs identified by performing a “jasmonic” keyword search in the <-1.1.#.ID-ANU.txt>.

<1.1.#.kinase.txt> IDs identified by performing a “kinase” keyword search in the <-1.1.#.ID-ANU.txt>.

<1.1.#.nodulin.txt> IDs identified by performing a “nodulin” keyword search in the <-1.1.#.ID-ANU.txt>.

<1.1.#.receptor.txt> IDs identified by performing a “receptor” keyword search in the <-1.1.#.ID-ANU.txt>.

<1.1.#.transcription factor.txt> IDs identified by performing a “transcription factor” and “transcription regulator” keywords search in the <-1.1.#.ID-ANU.txt>.

<1.1.#.transporter.txt> IDs identified by performing a “transporter” keyword search in the <-1.1.#.ID-ANU.txt>.

**Folder 1.2.Stage(s) specific transcript analysis**

<-1.2. Comparisons overview> describes the comparisons analysed in folder 1.2.

**Folder 1.2.# – Analysis of *L. japonicus* IDs regulated by *M. loti* specifically at particular time point(s) as mentioned in the file <-1.2. Comparisons overview>**

<-1.2.#.ID list> List of IDs and their corresponding M and FDR values in particular contrast(s) mentioned in the file <-1.2. Comparisons overview>.

<-1.2.#.ID-ANU.txt> putative annotation of the IDs found in the file <-1.2.#.ID list>.

<1.2.#.abscisic acid.txt> IDs identified by performing a “abscisic” keyword search in the <-1.2.#.ID-ANU.txt>.

<1.2.#.auxin.txt> IDs identified by performing a “auxin” keyword search in the <-1.2.#.ID-ANU.txt>.

<1.2.#.brassinosteroid.txt> IDs identified by performing a “brassinosteroid” and “BRASSINOSTEROID” keywords search in the <-1.2.#.ID-ANU.txt>.

<1.2.#.cytokinin.txt> IDs identified by performing a “cytokinin” keyword search in the <-1.2.#.ID-ANU.txt>.

<1.2.#.defense.txt> IDs identified by performing a “defense” keyword search in the <-1.2.#.ID-ANU.txt>.

<1.2.#.ethylene.txt> IDs identified by performing a “ethylene” keyword search in the <-1.2.#.ID-ANU.txt>.

<1.2.#.gibberellin.txt> IDs identified by performing a “gibberellin” keyword search in the <-1.2.#.ID-ANU.txt>.

<1.2.#.jasmonic acid.txt> IDs identified by performing a “jasmonic” keyword search in the <-1.2.#.ID-ANU.txt>.

<1.2.#.kinase.txt> IDs identified by performing a “kinase” keyword search in the <-1.2.#.ID-ANU.txt>.

<1.2.#.nodulin.txt> IDs identified by performing a “nodulin” keyword search in the <-1.2.#.ID-ANU.txt>.

<1.2.#.receptor.txt> IDs identified by performing a “receptor” keyword search in the <-1.2.#.ID-ANU.txt>.

<1.2.#.transcription factor.txt> IDs identified by performing a “transcription factor” and “transcription regulator” keywords search in the <-1.2.#.ID-ANU.txt>.

<1.2.#.transporter.txt> IDs identified by performing a “transporter” keyword search in the <-1.2.#.ID-ANU.txt>.

Experimental contrasts included in the statistical analysis

| **Inoculation_time_series** | | **All pairwise comparisons** | | |  |  |  |  |
| --- | --- | --- | --- | --- | --- | --- | --- | --- |
| *Treatment* | *Description* |  |  |  |  |  |  |  |
| T13 | WT_R7A1d_root | T13-T15 | T13-T34 | T13-T33 | T13-T32 | T13-T31 | T13-T23 | T13-T21 |
| T15 | WT_R7A3d_root |  | T15-T34 | T15-T33 | T15-T32 | T15-T31 | T15-T23 | T15-T21 |
| T34 | WT_R7A7d_root+nodule |  |  | T34-T33 | T34-T32 | T34-T31 | T34-T23 | T34-T21 |
| T33 | WT_R7A14d_nodule |  |  |  | T33-T32 | T33-T31 | T33-T23 | T33-T21 |
| T32 | WT_R7A21d_root+nodule |  |  |  |  | T32-T31 | T32-T23 | T32-T21 |
| T31 | WT_R7A21d_nodule |  |  |  |  |  | T31-T23 | T31-T21 |
| T23 | WT_nitrate3weeks_root |  |  |  |  |  |  | T23-T21 |
| T21 | WT_uninoculated_root |  |  |  |  |  |  |  |
