# Supplementary material for: Dissection of Symbiosis and Organ Development by Integrated Transcriptome Analysis of Lotus japonicus Mutant and Wild-Type Plants
Source: PLoS One. 2009 Aug 7;4(8):e6556. doi: 10.1371/journal.pone.0006556 (PMC2717213; doi:10.1371/journal.pone.0006556)
Supplement: Folder S2 — Inoculation 1 day (2.85 MB ZIP) [file pone.0006556.s010.zip › S2.INOCULATION 1 DAY copy/2.2.Specific transcript analysis copy/-2.2.Comparisons overview.doc]

| Contrast code | T05 - T11 | T13 - T21 | T14 - T21 | T06 - T11 |
| --- | --- | --- | --- | --- |
| Folder/Contrast | 1dpi *M.loti*-SZ /uninoc.SZ | 1dpi *M.loti*-whole root  /uninoc. whole root | 1dpi *M.loti nodC*-whole root  /uninoc. whole rootroot | 1dpi NF-SZ /uninoc.SZ |
| 2.2.1 |  |  |  |  |
| 2.2.2 |  |  |  |  |
| 2.2.3 |  |  |  |  |
| 2.2.4 |  |  |  |  |
| 2.2.5 |  |  |  |  |
| 2.2.6 |  |  |  |  |
| 2.2.7 |  |  |  |  |
| 2.2.8 |  |  |  |  |
| 2.2.9 |  |  |  |  |
| 2.2.10 |  |  |  |  |
| 2.2.11 |  |  |  |  |
| 2.2.12 |  |  |  |  |
| 2.2.13 |  |  |  |  |
| 2.2.14 |  |  |  |  |

Each folder (2.2.1. to 2.2.11) contains the analysis of *L. japonicus* IDs regulated by *M. loti*, *M. loti nod C* mutant bacteria or *M. loti* Nod factors (NF) in particular root sector(s). The transcript profile of the treated whole roots and susceptible zone (SZ)s were compared to the corresponding untreated root sectors. IDs that satisfied the established M and FDR criteria in the contrasts marked in green but not in those marked in red are listed in the file <-2.2.#. ID list>. No IDs were found in common for the contrasts mentioned in folders 2.2.3, 2.2.6, 2.2.11, 2.2.13 and 2.2.14 therefore they are empty.
