# Supplementary material for: Dissection of Symbiosis and Organ Development by Integrated Transcriptome Analysis of Lotus japonicus Mutant and Wild-Type Plants
Source: PLoS One. 2009 Aug 7;4(8):e6556. doi: 10.1371/journal.pone.0006556 (PMC2717213; doi:10.1371/journal.pone.0006556)
Supplement: Folder S2 — Inoculation 1 day (2.85 MB ZIP) [file pone.0006556.s010.zip › S2.INOCULATION 1 DAY copy/Supporting Folder 2 Info .doc]

Supporting Folder 2. **Inoculation 1 day**

**Folder 2.1.Total transcript analysis**

<-2.1. Comparisons overview> describes the comparisons analysed in folder 2.1.

**Folder 2.1.# – Analysis of *L. japonicus* IDs regulated 1 day upon treatment as mentioned in the file <-2.1. Comparisons overview>**

<-2.1.#.ID list> List of IDs and their corresponding M and FDR values in particular contrast(s) mentioned in the file <-2.1. Comparisons overview>.

<-2.1.#.ID-ANU.txt> putative annotation of the IDs found in the file <-2.1.#.ID list>.

<2.1.#.abscisic acid.txt> IDs identified by performing a “abscisic” keyword search in the <-2.1.#.ID-ANU.txt>.

<2.1.#.auxin.txt> IDs identified by performing a “auxin” keyword search in the <-2.1.#.ID-ANU.txt>.

<2.1.#.brassinosteroid.txt> IDs identified by performing a “brassinosteroid” and “BRASSINOSTEROID” keywords search in the <-2.1.#.ID-ANU.txt>.

<2.1.#.cytokinin.txt> IDs identified by performing a “cytokinin” keyword search in the <-2.1.#.ID-ANU.txt>.

<2.1.#.defense.txt> IDs identified by performing a “defense” keyword search in the <-2.1.#.ID-ANU.txt>.

<2.1.#.ethylene.txt> IDs identified by performing a “ethylene” keyword search in the <-2.1.#.ID-ANU.txt>.

<2.1.#.gibberellin.txt> IDs identified by performing a “gibberellin” keyword search in the <-2.1.#.ID-ANU.txt>.

<2.1.#.jasmonic acid.txt> IDs identified by performing a “jasmonic” keyword search in the <-2.1.#.ID-ANU.txt>.

<2.1.#.kinase.txt> IDs identified by performing a “kinase” keyword search in the <-2.1.#.ID-ANU.txt>.

<2.1.#.nodulin.txt> IDs identified by performing a “nodulin” keyword search in the <-2.1.#.ID-ANU.txt>.

<2.1.#.receptor.txt> IDs identified by performing a “receptor” keyword search in the <-2.1.#.ID-ANU.txt>.

<2.1.#.transcription factor.txt> IDs identified by performing a “transcription factor” and “transcription regulator” keywords search in the <-2.1.#.ID-ANU.txt>.

<2.1.#.transporter.txt> IDs identified by performing a “transporter” keyword search in the <-2.1.#.ID-ANU.txt>.

**Folder 2.2.Specific transcript analysis**

<-2.2. Comparisons overview> describes the comparisons analysed in folder 2.2.

**Folder 2.2.# – Analysis of *L. japonicus* IDs regulated 1 day after treatment specifically at particular root sector(s) as mentioned in the file <-2.2. Comparisons overview>**

<-2.2.#.ID list> List of IDs and their corresponding M and FDR values in particular contrast(s) mentioned in the file <-2.2. Comparisons overview>.

<-2.2.#.ID-ANU.txt> putative annotation of the IDs found in the file <-2.2.#.ID list>.

<2.2.#.abscisic acid.txt> IDs identified by performing a “abscisic” keyword search in the <-2.2.#.ID-ANU.txt>.

<2.2.#.auxin.txt> IDs identified by performing a “auxin” keyword search in the <-2.2.#.ID-ANU.txt>.

<2.2.#.brassinosteroid.txt> IDs identified by performing a “brassinosteroid” and “BRASSINOSTEROID” keywords search in the <-2.2.#.ID-ANU.txt>.

<2.2.#.cytokinin.txt> IDs identified by performing a “cytokinin” keyword search in the <-2.2.#.ID-ANU.txt>.

<2.2.#.defense.txt> IDs identified by performing a “defense” keyword search in the <-2.2.#.ID-ANU.txt>.

<2.2.#.ethylene.txt> IDs identified by performing a “ethylene” keyword search in the <-2.2.#.ID-ANU.txt>.

<2.2.#.gibberellin.txt> IDs identified by performing a “gibberellin” keyword search in the <-2.2.#.ID-ANU.txt>.

<2.2.#.jasmonic acid.txt> IDs identified by performing a “jasmonic” keyword search in the <-2.2.#.ID-ANU.txt>.

<2.2.#.kinase.txt> IDs identified by performing a “kinase” keyword search in the <-2.2.#.ID-ANU.txt>.

<2.2.#.nodulin.txt> IDs identified by performing a “nodulin” keyword search in the <-2.2.#.ID-ANU.txt>.

<2.2.#.receptor.txt> IDs identified by performing a “receptor” keyword search in the <-2.2.#.ID-ANU.txt>.

<2.2.#.transcription factor.txt> IDs identified by performing a “transcription factor” and “transcription regulator” keywords search in the <-2.2.#.ID-ANU.txt>.

<2.2.#.transporter.txt> IDs identified by performing a “transporter” keyword search in the <-2.2.#.ID-ANU.txt>.

Experimental contrasts included in the statistical analysis

| **Inoculation_1d** | | **All pairwise comparisons** | |  |
| --- | --- | --- | --- | --- |
| *Treatment* | *Description* |  |  |  |
| T05 | WT_R7A1d_SZ | T05 - T11 | T05 - T13 | T05 - T14 |
| T11 | WT_uninoculated_SZ |  |  |  |
| T13 | WT_R7A1d_root | T13 - T14 | T13 - T21 |  |
| T14 | WT_R7A-*nodC*_1d root | T14 - T21 |  |  |
| T21 | WT_uninoculated_root |  |  |  |
| T06 | WT_R7A-Nod Factor 1d_SZ | T06 - T11 |  |  |
