# Supplementary material for: Dissection of Symbiosis and Organ Development by Integrated Transcriptome Analysis of Lotus japonicus Mutant and Wild-Type Plants
Source: PLoS One. 2009 Aug 7;4(8):e6556. doi: 10.1371/journal.pone.0006556 (PMC2717213; doi:10.1371/journal.pone.0006556)
Supplement: Folder S3 — Nod-minus mutants (0.70 MB ZIP) [file pone.0006556.s011.zip › S3.NOD-MINUS MUTANTS copy/3.1. Nod minus vs wild-type total transcript analysis/-3.1.Comparisons overview.doc]

| Contrast code | T01-T07 | T02-T08 | T04-T10 | T03-T09 | T05-T11 |
| --- | --- | --- | --- | --- | --- |
| Folder/Contrast | *nfr1* inoc  /*nfr1* uninoc. | *nfr5* inoc  /*nfr5* uninoc. | *nup133* inoc  /*nup133* uninoc. | *nin* inoc  /*nin* uninoc. | wild-type inoc  /wild-type uninoc. |
| 3.1.1 |  | n.c.a. | n.c.a. | n.c.a. | n.c.a. |
| 3.1.2 | n.c.a. |  | n.c.a. | n.c.a. | n.c.a. |
| 3.1.3 | n.c.a. | n.c.a. |  | n.c.a. | n.c.a. |
| 3.1.4 | n.c.a. | n.c.a. | n.c.a. |  | n.c.a. |
| 3.1.5 | n.c.a. | n.c.a. | n.c.a. | n.c.a. |  |
| 3.1.6 |  |  | n.c.a. | n.c.a. | n.c.a. |
| 3.1.7 |  | n.c.a. |  | n.c.a. | n.c.a. |
| 3.1.8 |  | n.c.a. | n.c.a. |  | n.c.a. |
| 3.1.9 |  | n.c.a. | n.c.a. | n.c.a. |  |
| 3.1.10 | n.c.a. |  |  | n.c.a. | n.c.a. |
| 3.1.11 | n.c.a. |  | n.c.a. |  | n.c.a. |
| 3.1.12 | n.c.a. |  | n.c.a. | n.c.a. |  |
| 3.1.13 | n.c.a. | n.c.a. |  |  | n.c.a. |
| 3.1.14 | n.c.a. | n.c.a. |  | n.c.a. |  |
| 3.1.15 | n.c.a. | n.c.a. | n.c.a. |  |  |
| 3.1.16 |  |  |  | n.c.a. | n.c.a. |
| 3.1.17 |  |  | n.c.a. |  | n.c.a. |
| 3.1.18 |  |  | n.c.a. | n.c.a. |  |
| 3.1.19 | n.c.a. |  |  |  | n.c.a. |
| 3.1.20 | n.c.a. |  | n.c.a. |  |  |
| 3.1.21 | n.c.a. | n.c.a. |  |  |  |
| 3.1.22 |  | n.c.a. | n.c.a. |  |  |

Each folder (3.1.1. to 3.1.22) contains the analysis of *L. japonicus* IDs regulated by *M. loti* in wild-type and four nod minus mutants. The SZ transcript profile of each genotype was compared to the corresponding profile of uninoculated SZ. IDs that satisfied the established M and FDR criteria in the contrasts marked in green are listed in the file <-3.1.#. ID list>. No IDs were found in common for the contrasts mentioned in folders 3.1.7, 3.1.10 to 3.1.14, 3.1.16 to 3.1.21, and therefore they are empty.

n.c.a.-no criteria applied
