# Supplementary material for: Dissection of Symbiosis and Organ Development by Integrated Transcriptome Analysis of Lotus japonicus Mutant and Wild-Type Plants
Source: PLoS One. 2009 Aug 7;4(8):e6556. doi: 10.1371/journal.pone.0006556 (PMC2717213; doi:10.1371/journal.pone.0006556)
Supplement: Folder S3 — Nod-minus mutants (0.70 MB ZIP) [file pone.0006556.s011.zip › S3.NOD-MINUS MUTANTS copy/3.2. Nod minus vs wild-type specific transcript analysis/-3.2.Comparisons overview.doc]

| Contrast code | T01-T07 | T02-T08 | T04-T10 | T03-T09 | T05-T11 |
| --- | --- | --- | --- | --- | --- |
| Folder/Contrast | *nfr1* inoc  /*nfr1* uninoc. | *nfr5* inoc  /*nfr5* uninoc. | *nup133* inoc  /*nup133* uninoc. | *nin* inoc  /*nin* uninoc. | wild-type inoc  /wild-type uninoc. |
| 3.2.1 |  |  |  |  |  |
| 3.2.2 |  |  |  |  |  |
| 3.2.3 |  |  |  |  |  |
| 3.2.4 |  |  |  |  |  |
| 3.2.5 |  |  |  |  |  |
| 3.2.6 |  |  |  |  |  |
| 3.2.7 |  |  |  |  |  |
| 3.2.8 |  |  |  |  |  |
| 3.2.9 |  |  |  |  |  |
| 3.2.10 |  |  |  |  |  |
| 3.2.11 |  |  |  |  |  |
| 3.2.12 |  |  |  |  |  |
| 3.2.13 |  |  |  |  |  |
| 3.2.14 |  |  |  |  |  |
| 3.2.15 |  |  |  |  |  |
| 3.2.16 |  |  |  |  |  |
| 3.2.17 |  |  |  |  |  |
| 3.2.18 |  |  |  |  |  |
| 3.2.19 |  |  |  |  |  |
| 3.2.20 |  |  |  |  |  |
| 3.2.21 |  |  |  |  |  |
| 3.2.22 |  |  |  |  |  |

Each folder (3.2.1. to 3.2.22) contains the analysis of *L. japonicus* IDs regulated by *M. loti* in wild-type and four nod minus mutants. The SZ transcript profile of each genotype was compared to the corresponding profile of uninoculated SZ. IDs that satisfied the established M and FDR criteria in the contrasts marked in green but not in those marked in red are listed in the file <-3.2.#. ID list>. No IDs were found in common for the contrasts mentioned in folders 3.2.2, 3.2.7, 3.2.8, 3.2.10 to 3.2.14, 3.2.16 to 3.2.21, and therefore they are empty.
