# Supplementary material for: Dissection of Symbiosis and Organ Development by Integrated Transcriptome Analysis of Lotus japonicus Mutant and Wild-Type Plants
Source: PLoS One. 2009 Aug 7;4(8):e6556. doi: 10.1371/journal.pone.0006556 (PMC2717213; doi:10.1371/journal.pone.0006556)
Supplement: Folder S3 — Nod-minus mutants (0.70 MB ZIP) [file pone.0006556.s011.zip › S3.NOD-MINUS MUTANTS copy/Supporting Folder 3 Info.doc]

Supporting Folder 3. **Nod-minus mutants**

**Folder 3.1.Nod minus vs wild-type total transcript analysis**

<-3.1. Comparisons overview> describes the comparisons analysed in folder 3.1.

**Folder 3.1.# – Analysis of *L. japonicus* IDs regulated by *M. loti* in particular nod-minus mutant(s) as mentioned in the file <-3.1. Comparisons overview>**

<-3.1.#.ID list> List of IDs and their corresponding M and FDR values in particular contrast(s) mentioned in the file <-3.1. Comparisons overview>.

<-3.1.#.ID-ANU.txt> putative annotation of the IDs found in the file <-3.1.#.ID list>.

<3.1.#.abscisic acid.txt> IDs identified by performing a “abscisic” keyword search in the <-3.1.#.ID-ANU.txt>.

<3.1.#.auxin.txt> IDs identified by performing a “auxin” keyword search in the <-3.1.#.ID-ANU.txt>.

<3.1.#.brassinosteroid.txt> IDs identified by performing a “brassinosteroid” and “BRASSINOSTEROID” keywords search in the <-3.1.#.ID-ANU.txt>.

<3.1.#.cytokinin.txt> IDs identified by performing a “cytokinin” keyword search in the <-3.1.#.ID-ANU.txt>.

<3.1.#.defense.txt> IDs identified by performing a “defense” keyword search in the <-3.1.#.ID-ANU.txt>.

<3.1.#.ethylene.txt> IDs identified by performing a “ethylene” keyword search in the <-3.1.#.ID-ANU.txt>.

<3.1.#.gibberellin.txt> IDs identified by performing a “gibberellin” keyword search in the <-3.1.#.ID-ANU.txt>.

<3.1.#.jasmonic acid.txt> IDs identified by performing a “jasmonic” keyword search in the <-3.1.#.ID-ANU.txt>.

<3.1.#.kinase.txt> IDs identified by performing a “kinase” keyword search in the <-3.1.#.ID-ANU.txt>.

<3.1.#.nodulin.txt> IDs identified by performing a “nodulin” keyword search in the <-3.1.#.ID-ANU.txt>.

<3.1.#.receptor.txt> IDs identified by performing a “receptor” keyword search in the <-3.1.#.ID-ANU.txt>.

<3.1.#.transcription factor.txt> IDs identified by performing a “transcription factor” and “transcription regulator” keywords search in the <-3.1.#.ID-ANU.txt>.

<3.1.#.transporter.txt> IDs identified by performing a “transporter” keyword search in the <-3.1.#.ID-ANU.txt>.

**Folder 3.2. Nod minus vs wild-type specific transcript analysis**

<-3.2. Comparisons overview> describes the comparisons analysed in folder 3.2.

**Folder 3.2.# – Analysis of *L. japonicus* IDs regulated by *M. loti* specifically at particular time point(s) as mentioned in the file <-3.2. Comparisons overview>**

<-3.2.#.ID list> List of IDs and their corresponding M and FDR values in particular contrast(s) mentioned in the file <-3.2. Comparisons overview>.

<-3.2.#.ID-ANU.txt> putative annotation of the IDs found in the file <-3.2.#.ID list>.

<3.2.#.abscisic acid.txt> IDs identified by performing a “abscisic” keyword search in the <-3.2.#.ID-ANU.txt>.

<3.2.#.auxin.txt> IDs identified by performing a “auxin” keyword search in the <-3.2.#.ID-ANU.txt>.

<3.2.#.brassinosteroid.txt> IDs identified by performing a “brassinosteroid” and “BRASSINOSTEROID” keywords search in the <-3.2.#.ID-ANU.txt>.

<3.2.#.cytokinin.txt> IDs identified by performing a “cytokinin” keyword search in the <-3.2.#.ID-ANU.txt>.

<3.2.#.defense.txt> IDs identified by performing a “defense” keyword search in the <-3.2.#.ID-ANU.txt>.

<3.2.#.ethylene.txt> IDs identified by performing a “ethylene” keyword search in the <-3.2.#.ID-ANU.txt>.

<3.2.#.gibberellin.txt> IDs identified by performing a “gibberellin” keyword search in the <-3.2.#.ID-ANU.txt>.

<3.2.#.jasmonic acid.txt> IDs identified by performing a “jasmonic” keyword search in the <-3.2.#.ID-ANU.txt>.

<3.2.#.kinase.txt> IDs identified by performing a “kinase” keyword search in the <-3.2.#.ID-ANU.txt>.

<3.2.#.nodulin.txt> IDs identified by performing a “nodulin” keyword search in the <-3.2.#.ID-ANU.txt>.

<3.2.#.receptor.txt> IDs identified by performing a “receptor” keyword search in the <-3.2.#.ID-ANU.txt>.

<3.2.#.transcription factor.txt> IDs identified by performing a “transcription factor” and “transcription regulator” keywords search in the <-3.2.#.ID-ANU.txt>.

<3.2.#.transporter.txt> IDs identified by performing a “transporter” keyword search in the <-3.2.#.ID-ANU.txt>.

Experimental contrasts included in the statistical analysis

| *Treatment* | *Description* | *Genotype* | *Treatment* | *Interaction* |
| --- | --- | --- | --- | --- |
| T01 | *nfr1*_R7A1d_SZ | T01-T05 | T01-T07 | (T05-T11)-(T01-T07) |
| T02 | *nfr5*_R7A1d_SZ | T02-T05 | T02-T08 | (T05-T11)-(T02-T08) |
| T03 | *nin*_R7A1d_SZ | T03-T05 | T03-T09 | (T05-T11)-(T03-T09) |
| T04 | *nup133*_R7A1d_SZ | T04-T05 | T04-T10 | (T05-T11)-(T04-T10) |
| T05 | WT_R7A1d_SZ | T05-T11 | T05-T06 |  |
| T06 | WT_R7A Nod Factor 1d_SZ |  | T06-T11 |  |
| T07 | *nfr1*_uninoculated_SZ | T07-T11 |  | (T01-T07)-(T02-T08) |
| T08 | *nfr5*_uninoculated_SZ | T08-T11 |  | (T01-T07)-(T03-T09) |
| T09 | *nin*_uninoculated_SZ | T09-T11 |  | (T01-T07)-(T04-T10) |
| T10 | *nup133*_uninoculated_SZ | T10-T11 |  | (T02-T08)-(T03-T09) |
| T11 | WT_uninoculated_SZ |  |  | (T02-T08)-(T04-T10) |
|  |  |  |  | (T03-T09)-(T04-T10) |
