# Supplementary material for: Dissection of Symbiosis and Organ Development by Integrated Transcriptome Analysis of Lotus japonicus Mutant and Wild-Type Plants
Source: PLoS One. 2009 Aug 7;4(8):e6556. doi: 10.1371/journal.pone.0006556 (PMC2717213; doi:10.1371/journal.pone.0006556)
Supplement: Folder S4 — Cyclops mutant (0.59 MB ZIP) [file pone.0006556.s012.zip › S4.CYCLOPS MUTANT copy/4.1. Cyclops vs wild type specific transcript/-4.1.Comparisons overview.doc]

| Contrast code | T28 - T25 | T13 - T21 | T15 - T21 | T34 - T21 | T32 - T21 |
| --- | --- | --- | --- | --- | --- |
| Folder/Contrast | *cyclops* 21dpi/  *cyclops* uninoc. | Wt 1dpi/uninoc. | Wt 3dpi/uninoc. | Wt 7dpi/uninoc. | Wt 21dpi/uninoc. |
| 4.1.1 |  |  |  |  |  |
| 4.1.2 |  |  |  |  |  |
| 4.1.3 |  |  |  |  |  |
| 4.1.4 |  |  |  |  |  |
| 4.1.5 |  |  |  |  |  |
| 4.1.6 |  |  |  |  |  |
| 4.1.7 |  |  |  |  |  |
| 4.1.8 |  |  |  |  |  |
| 4.1.9 |  |  |  |  |  |
| 4.1.10 |  |  |  |  |  |
| 4.1.11 |  |  |  |  |  |
| 4.1.12 |  |  |  |  |  |
| 4.1.13 |  |  |  |  |  |

Each folder (4.1.1. to 4.1.13) contains the analysis of *L. japonicus* IDs regulated by *M. loti* in wild type plants at particular time point(s) and in *cyclops* at 21dpi. The whole root transcript profile of each developmental stage (1dpi, 3dpi, 7dpi, 21dpi) was compared to the profile of corresponding uninoculated roots. IDs that satisfied the established M and FDR criteria in the contrasts marked in green but not in those marked in red are listed in the file <-4.1.#. ID list>.
