# Supplementary material for: Dissection of Symbiosis and Organ Development by Integrated Transcriptome Analysis of Lotus japonicus Mutant and Wild-Type Plants
Source: PLoS One. 2009 Aug 7;4(8):e6556. doi: 10.1371/journal.pone.0006556 (PMC2717213; doi:10.1371/journal.pone.0006556)
Supplement: Folder S4 — Cyclops mutant (0.59 MB ZIP) [file pone.0006556.s012.zip › S4.CYCLOPS MUTANT copy/4.2. Cyclops vs nin vs wild type/-4.2.Comparisons overview.doc]

| Contrast code | T03 - T09 | T05 - T11 | T13 - T21 | T28 - T25 |
| --- | --- | --- | --- | --- |
| Folder/Contrast | *nin* 1dpi SZ/  *nin* uninoc. | wt-SZ-1dpi/  wt uninoc. | wt-WR-1dpi/  wt uninoc. | *cyclops* 21dpi/  *cyclops* uninoc. |
| 4.2.1 |  |  |  |  |
| 4.2.2 |  |  |  |  |
| 4.2.3 |  |  |  |  |
| 4.2.4 |  |  |  |  |
| 4.2.5 |  |  |  |  |
| 4.2.6 |  |  | *n.c.a.* | *n.c.a.* |
| 4.2.7 | *n.c.a.* | *n.c.a.* |  |  |
| 4.2.8 | *n.c.a.* |  | *n.c.a.* |  |
| 4.2.9 |  | *n.c.a.* |  | *n.c.a.* |

Each folder (4.2.1. to 4.2.9) contains the analysis of *L. japonicus* IDs regulated by *M. loti* in wild type susceptible zone (SZ) and whole root (WR) at 1dpi, in *nin* SZ at 1dpi and in *cyclops* WR at 21dpi. The specified root transcript profile was compared to the profile of the corresponding uninoculated root sectors. IDs that satisfied the established M and FDR criteria in the contrasts marked in green but not in those marked in red are listed in the file <-4.2.#. ID list>.

*n.c.a.* – no criteria applied.
