# Supplementary material for: Dissection of Symbiosis and Organ Development by Integrated Transcriptome Analysis of Lotus japonicus Mutant and Wild-Type Plants
Source: PLoS One. 2009 Aug 7;4(8):e6556. doi: 10.1371/journal.pone.0006556 (PMC2717213; doi:10.1371/journal.pone.0006556)
Supplement: Folder S5 — Fix-minus mutants (9.19 MB ZIP) [file pone.0006556.s013.zip › S5.FIX-MINUS MUTANTS copy/5.1.Fix_minus_Nodule_vs_Uninoclated_Root/5.1.2. Specific transcript analysis/-5.1.2.Comparisons overview.doc]

| Constrast code | T29 - T26 | T30 - T27 | T33 - T21 | T31 - T21 |
| --- | --- | --- | --- | --- |
| Folder/Contrast | *sen1*nod/unin | *sst1*nod/unin | Wt14dnod/unin | Wt21dnod/unin |
| 5.1.2.1 |  |  |  |  |
| 5.1.2.2 |  |  |  |  |
| 5.1.2.3 |  |  |  |  |
| 5.1.2.4 |  |  |  |  |
| 5.1.2.5 |  |  |  |  |
| 5.1.2.6 |  |  |  |  |
| 5.1.2.7 |  |  |  |  |
| 5.1.2.8 |  |  |  |  |
| 5.1.2.9 |  |  |  |  |
| 5.1.2.10 |  |  |  |  |
| 5.1.2.11 |  |  |  |  |
| 5.1.2.12 |  |  |  |  |
| 5.1.2.13 |  |  |  |  |
| 5.1.2.14 |  |  |  |  |
| 5.1.2.15 |  |  |  |  |

Each folder (5.1.2.1. to 5.1.2.15) contains the analysis of *L. japonicus* IDs regulated in the *Ljsen1* and *Ljsst1* mutants and wild type 14 and 21 dpi. The nodules were compared to the profile of the corrosponding uninoculated roots. IDs that satisfied the established M and FDR criteria in the contrasts marked in green but not in those marked in red are listed in the file <-5.1.2.#. ID list>.
