# Supplementary material for: Dissection of Symbiosis and Organ Development by Integrated Transcriptome Analysis of Lotus japonicus Mutant and Wild-Type Plants
Source: PLoS One. 2009 Aug 7;4(8):e6556. doi: 10.1371/journal.pone.0006556 (PMC2717213; doi:10.1371/journal.pone.0006556)
Supplement: Folder S5 — Fix-minus mutants (9.19 MB ZIP) [file pone.0006556.s013.zip › S5.FIX-MINUS MUTANTS copy/5.2.Fix_Minus_Nodule_vs_Wt_Nodule/5.2.2.Specific transcript analysis/-5.2.2.Comparisons overview.doc]

| Contrast code | T29 - T31 | T30 - T31 |
| --- | --- | --- |
| Folder/Contrast | *sen1*nod/wtnod | *sst1*nod/wtnod |
| 5.2.2.1 |  |  |
| 5.2.2.2 |  |  |
| 5.2.2.3 |  |  |

Each folder (5.2.2.1. to 5.2.2.3) contains the analysis of *L. japonicus* IDs regulated in *Ljsen1* and *Ljsst1* mutants. The fixation minus mutant nodules were compared to the profile of wild type nodule2 21 dpi. IDs that satisfied the established M and FDR criteria in the contrasts marked in green but not in those marked in red are listed in the file <-5.2.2.#. ID list>.
