# Supplementary material for: Dissection of Symbiosis and Organ Development by Integrated Transcriptome Analysis of Lotus japonicus Mutant and Wild-Type Plants
Source: PLoS One. 2009 Aug 7;4(8):e6556. doi: 10.1371/journal.pone.0006556 (PMC2717213; doi:10.1371/journal.pone.0006556)
Supplement: Folder S5 — Fix-minus mutants (9.19 MB ZIP) [file pone.0006556.s013.zip › S5.FIX-MINUS MUTANTS copy/Supporting Folder 5 Info .doc]

Supporting Folder **5.Fix-minus mutants**

**5.1.Fixation minus mutant nodules and wild type nodules 14dpi and 21 dpi compared to their respective uninoculated root.**

**5.1.1.Total transcript analysis**

<-5.1.1.Comparisons overview> describes the comparisons analysed in folder 5.1.1

**5.1.1# – Analysis of *L. japonicus* IDs regulated in fixation minus mutant and wild type nodules when compared to uninoculated roots as mentioned in the file <-5.1.1. Comparisons overview>**

<-5.1.1.#.ID list> List of IDs and their corresponding M and FDR values in particular contrast(s) mentioned in the file <-5.1.1. Comparisons overview>.

<-5.1.1.#.ID-ANU.txt> putative annotation of the IDs found in the file <-5.1.1.#.ID list>.

<5.1.1.#.abscisic acid.txt> IDs identified by performing a “abscisic” keyword search in the <-5.1.1.#.ID-ANU.txt>.

<5.1.1.#.auxin.txt> IDs identified by performing a “auxin” keyword search in the <-5.1.1.#.ID-ANU.txt>.

<5.1.1.#.brassinosteroid.txt> IDs identified by performing a “brassinosteroid” and “BRASSINOSTEROID” keywords search in the <-5.1.1.#.ID-ANU.txt>.

<5.1.1.#.cytokinin.txt> IDs identified by performing a “cytokinin” keyword search in the <-5.1.1.#.ID-ANU.txt>.

<5.1.1.#.defense.txt> IDs identified by performing a “defense” keyword search in the <-5.1.1.#.ID-ANU.txt>.

<5.1.1.#.ethylene.txt> IDs identified by performing a “ethylene” keyword search in the <-5.1.1.#.ID-ANU.txt>.

<5.1.1.#.gibberellin.txt> IDs identified by performing a “gibberellin” keyword search in the <-5.1.1.#.ID-ANU.txt>.

<5.1.1.#.jasmonic acid.txt> IDs identified by performing a “jasmonic” keyword search in the <-5.1.1.#.ID-ANU.txt>.

<5.1.1.#.kinase.txt> IDs identified by performing a “kinase” keyword search in the <-5.1.1.#.ID-ANU.txt>.

<5.1.1.#.nodulin.txt> IDs identified by performing a “nodulin” keyword search in the <-5.1.1.#.ID-ANU.txt>.

<5.1.1.#.receptor.txt> IDs identified by performing a “receptor” keyword search in the <-5.1.1.#.ID-ANU.txt>.

<5.1.1.#.transcription factor.txt> IDs identified by performing a “transcription factor” keyword search in the <-5.1.1.#.ID-ANU.txt>.

<5.1.1.#.transporter.txt> IDs identified by performing a “transporter” keyword search in the <-5.1.1.#.ID-ANU.txt>.

**5.1.2.Specific transcript analysis**

<-5.1.2. Comparisons overview> describes the comparisons analysed in folder 5.1.2.

**5.1.2.# – Analysis of *L. japonicus* IDs regulated specifically in fixation minus mutant and wild type nodules when compared to uninoculated roots as mentioned in the file <-5.1.2. Comparisons overview>**

<-5.1.2.#.ID list> List of IDs and their corresponding M and FDR values in particular contrast(s) mentioned in the file <-5.1.2. Comparisons overview>.

<-5.1.2.#.ID-ANU.txt> putative annotation of the IDs found in the file <-5.1.2.#.ID list>.

<5.1.2.#.abscisic acid.txt> IDs identified by performing a “abscisic” keyword search in the <-5.1.2.#.ID-ANU.txt>.

<5.1.2.#.auxin.txt> IDs identified by performing a “auxin” keyword search in the <-5.1.2.#.ID-ANU.txt>.

<5.1.2.#.brassinosteroid.txt> IDs identified by performing a “brassinosteroid” and “BRASSINOSTEROID” keywords search in the <-5.1.2.#.ID-ANU.txt>.

<5.1.2.#.cytokinin.txt> IDs identified by performing a “cytokinin” keyword search in the <-5.1.2.#.ID-ANU.txt>.

<5.1.2.#.defense.txt> IDs identified by performing a “defense” keyword search in the <-5.1.2.#.ID-ANU.txt>.

<5.1.2.#.ethylene.txt> IDs identified by performing a “ethylene” keyword search in the <-5.1.2.#.ID-ANU.txt>.

<5.1.2.#.gibberellin.txt> IDs identified by performing a “gibberellin” keyword search in the <-5.1.2.#.ID-ANU.txt>.

<5.1.2.#.jasmonic acid.txt> IDs identified by performing a “jasmonic” keyword search in the <-5.1.2.#.ID-ANU.txt>.

<5.1.2.#.kinase.txt> IDs identified by performing a “kinase” keyword search in the <-5.1.2.#.ID-ANU.txt>.

<5.1.2.#.nodulin.txt> IDs identified by performing a “nodulin” keyword search in the <-5.1.2.#.ID-ANU.txt>.

<5.1.2.#.receptor.txt> IDs identified by performing a “receptor” keyword search in the <-5.1.2.#.ID-ANU.txt>.

<5.1.2.#.transcription factor.txt> IDs identified by performing a “transcription factor” keyword search in the <-5.1.2.#.ID-ANU.txt>.

<5.1.2.#.transporter.txt> IDs identified by performing a “transporter” keyword search in the <-5.1.2.#.ID-ANU.txt>.

**5.2.Fixation minus mutant nodules compared to wild type nodule 21dpi.**

**5.2.1.Total transcript analysis**

<-5.2.1.Comparisons overview> describes the comparisons analysed in folder 5.2.1

**5.2.1# – Analysis of *L. japonicus* IDs regulated in fixation minus mutant nodules when compared to wild type nodules 21 dpi <-5.2.1. Comparisons overview>**

<-5.2.1.#.ID list> List of IDs and their corresponding M and FDR values in particular contrast(s) mentioned in the file <-5.2.1. Comparisons overview>.

<-5.2.1.#.ID-ANU.txt> putative annotation of the IDs found in the file <-5.2.1.#.ID list>.

<5.2.1.#.abscisic acid.txt> IDs identified by performing a “abscisic” keyword search in the <-5.2.1.#.ID-ANU.txt>.

<5.2.1.#.auxin.txt> IDs identified by performing a “auxin” keyword search in the <-5.2.1.#.ID-ANU.txt>.

<5.2.1.#.brassinosteroid.txt> IDs identified by performing a “brassinosteroid” and “BRASSINOSTEROID” keywords search in the <-5.2.1.#.ID-ANU.txt>.

<5.2.1.#.cytokinin.txt> IDs identified by performing a “cytokinin” keyword search in the <-5.2.1.#.ID-ANU.txt>.

<5.2.1.#.defense.txt> IDs identified by performing a “defense” keyword search in the <-5.2.1.#.ID-ANU.txt>.

<5.2.1.#.ethylene.txt> IDs identified by performing a “ethylene” keyword search in the <-5.2.1.#.ID-ANU.txt>.

<5.2.1.#.gibberellin.txt> IDs identified by performing a “gibberellin” keyword search in the <-5.2.1.#.ID-ANU.txt>.

<5.2.1.#.jasmonic acid.txt> IDs identified by performing a “jasmonic” keyword search in the <-5.2.1.#.ID-ANU.txt>.

<5.2.1.#.kinase.txt> IDs identified by performing a “kinase” keyword search in the <-5.2.1.#.ID-ANU.txt>.

<5.2.1.#.nodulin.txt> IDs identified by performing a “nodulin” keyword search in the <-5.2.1.#.ID-ANU.txt>.

<5.2.1.#.receptor.txt> IDs identified by performing a “receptor” keyword search in the <-5.2.1.#.ID-ANU.txt>.

<5.2.1.#.transcription factor.txt> IDs identified by performing a “transcription factor” keyword search in the <-5.2.1.#.ID-ANU.txt>.

<5.2.1.#.transporter.txt> IDs identified by performing a “transporter” keyword search in the <-5.2.1.#.ID-ANU.txt>.

**5.2.2.Specific transcript analysis**

<-5.2.2. Comparisons overview> describes the comparisons analysed in folder 5.2.2.

**5.2.2.# – Analysis of *L. japonicus* IDs regulated specifically in fixation minus mutant nodules when compared to wild type nodules 21 dpi <-5.2.2. Comparisons overview>**

<-5.2.2.#.ID list> List of IDs and their corresponding M and FDR values in particular contrast(s) mentioned in the file <-5.2.2. Comparisons overview>.

<-5.2.2.#.ID-ANU.txt> putative annotation of the IDs found in the file <-5.2.2.#.ID list>.

<5.2.2.#.abscisic acid.txt> IDs identified by performing a “abscisic” keyword search in the <-5.2.2.#.ID-ANU.txt>.

<5.2.2.#.auxin.txt> IDs identified by performing a “auxin” keyword search in the <-5.2.2.#.ID-ANU.txt>.

<5.2.2.#.brassinosteroid.txt> IDs identified by performing a “brassinosteroid” and “BRASSINOSTEROID” keywords search in the <-5.2.2.#.ID-ANU.txt>.

<5.2.2.#.cytokinin.txt> IDs identified by performing a “cytokinin” keyword search in the <-5.2.2.#.ID-ANU.txt>.

<5.2.2.#.defense.txt> IDs identified by performing a “defense” keyword search in the <-5.2.2.#.ID-ANU.txt>.

<5.2.2.#.ethylene.txt> IDs identified by performing a “ethylene” keyword search in the <-5.2.2.#.ID-ANU.txt>.

<5.2.2.#.gibberellin.txt> IDs identified by performing a “gibberellin” keyword search in the <-5.2.2.#.ID-ANU.txt>.

<5.2.2.#.jasmonic acid.txt> IDs identified by performing a “jasmonic” keyword search in the <-5.2.2.#.ID-ANU.txt>.

<5.2.2.#.kinase.txt> IDs identified by performing a “kinase” keyword search in the <-5.2.2.#.ID-ANU.txt>.

<5.2.2.#.nodulin.txt> IDs identified by performing a “nodulin” keyword search in the <-5.2.2.#.ID-ANU.txt>.

<5.2.2.#.receptor.txt> IDs identified by performing a “receptor” keyword search in the <-5.2.2.#.ID-ANU.txt>.

<5.2.2.#.transcription factor.txt> IDs identified by performing a “transcription factor” keyword search in the <-5.2.2.#.ID-ANU.txt>.

<5.2.2.#.transporter.txt> IDs identified by performing a “transporter” keyword search in the <-5.2.2.#.ID-ANU.txt>.

Experimental contrasts included in the analysis

| *Treatment* | *Description* | *Genotype* | *Treatment* | *Interaction* |
| --- | --- | --- | --- | --- |
| T25 | *cyclops*_uninoculated | T25-T21 |  |  |
| T26 | *sen1*_uninoculated | T26-T21 |  |  |
| T27 | *sst1*_uninoculated | T27-T21 |  |  |
| T21 | WT_uninoculated_root |  |  |  |
| T28 | *cyclops*_R7A21d_root+nodule | T28-T31 | T28-T25 | (T31-T21)-(T28-T25) |
| T29 | *sen1*_R7A21d_nodule | T29-T31 | T29-T26 | (T31-T21)-(T29-T26) |
| T30 | *sst1*_R7A21d_nodule | T30-T31 | T30-T27 | (T31-T21)-(T30-T27) |
| T31 | WT_R7A21d_nodule |  | T31-T21 | (T29-T26)-(T30-T27) |
| T33 | WT_R7A14d_nodule | T33 - T21 |  |  |
