# Supplementary material for: Dissection of Symbiosis and Organ Development by Integrated Transcriptome Analysis of Lotus japonicus Mutant and Wild-Type Plants
Source: PLoS One. 2009 Aug 7;4(8):e6556. doi: 10.1371/journal.pone.0006556 (PMC2717213; doi:10.1371/journal.pone.0006556)
Supplement: Folder S6 — Organs (0.23 MB ZIP) [file pone.0006556.s014.zip › S6.ORGANS copy/Supporting Folder 6 Info.doc]

Supporting Folder 6. **Organs**

**Folder 6.# – Analysis of *L. japonicus* organ (#) specific IDs**

<-#.ID list> List of # specific IDs

<#_ANU.txt> putative annotation of the IDs found in the file <#.ID list>.

<#.abscisic acid.txt> IDs identified by performing a “abscisic” keyword search in the <#._ANU.txt>.

<#auxin.txt> IDs identified by performing a “auxin” keyword search in the <#._ANU.txt>.

<#brassinosteroid.txt> IDs identified by performing a “brassinosteroid” and “BRASSINOSTEROID” keywords search in the <#._ANU.txt>.

<#cytokinin.txt> IDs identified by performing a “cytokinin” keyword search in the <#._ANU.txt>.

<#defense.txt> IDs identified by performing a “defense” keyword search in the <#._ANU.txt>.

<#ethylene.txt> IDs identified by performing a “ethylene” keyword search in the <#._ANU.txt>.

<#gibberellin.txt> IDs identified by performing a “gibberellin” keyword search in the <#._ANU.txt>.

<#jasmonic acid.txt> IDs identified by performing a “jasmonic” keyword search in the <#._ANU.txt>.

<#kinase.txt> IDs identified by performing a “kinase” keyword search in the <#._ANU.txt>.

<#nodulin.txt> IDs identified by performing a “nodulin” keyword search in the <#._ANU.txt>.

<#receptor.txt> IDs identified by performing a “receptor” keyword search in the <#._ANU.txt>.

<#transcription factor.txt> IDs identified by performing a “transcription factor” and “transcription regulator” keywords search in the <#._ANU.txt>.

<#transporter.txt> IDs identified by performing a “transporter” keyword search in the <#._ANU.txt>.
