# Supplementary material for: Dissection of Symbiosis and Organ Development by Integrated Transcriptome Analysis of Lotus japonicus Mutant and Wild-Type Plants
Source: PLoS One. 2009 Aug 7;4(8):e6556. doi: 10.1371/journal.pone.0006556 (PMC2717213; doi:10.1371/journal.pone.0006556)
Supplement: Folder S7 — Nitrate (0.85 MB ZIP) [file pone.0006556.s015.zip › S7.NITRATE copy/7.1.Root-nitrate vs rhizobium/7.1.1.Root total transcript analysis/-7.1.1.Comparisons overview.doc]

| Contrast code | T13 - T21 | T15 - T21 | T34 - T21 | T32 - T21 | T23-T21 |
| --- | --- | --- | --- | --- | --- |
| Folder/Contrast | 1dpi/uninoc. | 3dpi/uninoc. | 7dpi/uninoc. | 21dpi/uninoc. | 21d NO_3_/uninoc. |
| 7.1.1.1 |  | n.c.a. | n.c.a. | n.c.a. |  |
| 7.1.1.2 | n.c.a. |  | n.c.a. | n.c.a. |  |
| 7.1.1.3 | n.c.a. | n.c.a. |  | n.c.a. |  |
| 7.1.1.4 | n.c.a. | n.c.a. | n.c.a. |  |  |
| 7.1.1.5 |  |  | n.c.a. | n.c.a. |  |
| 7.1.1.6 | n.c.a. |  |  | n.c.a. |  |
| 7.1.1.7 | n.c.a. | n.c.a. |  |  |  |
| 7.1.1.8 |  | n.c.a. |  | n.c.a. |  |
| 7.1.1.9 | n.c.a. |  | n.c.a. |  |  |
| 7.1.1.10 |  |  |  |  | n.c.a. |
| 7.1.1.11 | n.c.a. | n.c.a. | n.c.a. | n.c.a. |  |

Each folder (7.1.1.1. to 7.1.1.11) contains the analysis of *L. japonicus* IDs regulated by *M. loti* at particular time point(s) and/or nitrate (21 days). The whole root transcript profile of each developmental stage (1dpi, 3dpi, 7dpi, 21dpi) or treatment was compared to the profile of uninoculated roots and IDs that satisfied the established M and FDR criteria in the contrasts marked in green are listed in the file <-7.1.1.#. ID list>.

n.c.a.-no criteria applied.
