# Supplementary material for: Dissection of Symbiosis and Organ Development by Integrated Transcriptome Analysis of Lotus japonicus Mutant and Wild-Type Plants
Source: PLoS One. 2009 Aug 7;4(8):e6556. doi: 10.1371/journal.pone.0006556 (PMC2717213; doi:10.1371/journal.pone.0006556)
Supplement: Folder S7 — Nitrate (0.85 MB ZIP) [file pone.0006556.s015.zip › S7.NITRATE copy/7.2.Nitrate-root vs shoot/-7.2.Comparisons overview.doc]

| Contrast code | T23-T21 | T24-T22 |
| --- | --- | --- |
| Folder/Contrast | NO_3_ root/  uninoc. root | NO_3_ shoot/  uninoc. shoot |
| 7.2.1 |  |  |
| 7.2.2 |  |  |
| 7.2.3 |  |  |

Each folder (7.2.1. to 7.2.3) contains the analysis of *L. japonicus* IDs regulated by nitrate in the root and shoot. The whole root or shoot transcript profile was compared to the profile of uninoculated root or shoot. IDs that satisfied the established M and FDR criteria in the contrasts marked in green but not in those marked in red are listed in the file <-7.2.#. ID list>.
